# FOXM1 Upregulation Is an Early Event in Human Squamous Cell Carcinoma and it Is Enhanced by Nicotine during Malignant Transformation

**DOI:** 10.1371/journal.pone.0004849

**Published:** 2009-03-16

**Authors:** Emilios Gemenetzidis, Amrita Bose, Adeel M. Riaz, Tracy Chaplin, Bryan D. Young, Muhammad Ali, David Sugden, Johanna K. Thurlow, Sok-Ching Cheong, Soo-Hwang Teo, Hong Wan, Ahmad Waseem, Eric K. Parkinson, Farida Fortune, Muy-Teck Teh

**Affiliations:** 1 Centre for Clinical and Diagnostic Oral Sciences, Institute of Dentistry, Barts & The London School of Medicine and Dentistry, Queen Mary University of London, London, United Kingdom; 2 Cancer Research UK Medical Oncology Laboratory, Institute of Dentistry, Barts & The London School of Medicine and Dentistry, Queen Mary University of London, London, United Kingdom; 3 Division of Reproduction and Endocrinology, School of Biomedical and Health Sciences, King's College London, London, United Kingdom; 4 The Beatson Institute for Cancer Research, Glasgow, United Kingdom; 5 Cancer Research Initiatives Foundation (CARIF), 2nd Floor Outpatient Centre, Subang Jaya Medical Centre, Selangor, Malaysia; University of Hong Kong, Hong Kong

## Abstract

**Background:**

Cancer associated with smoking and drinking remains a serious health problem worldwide. The survival of patients is very poor due to the lack of effective early biomarkers. FOXM1 overexpression is linked to the majority of human cancers but its mechanism remains unclear in head and neck squamous cell carcinoma (HNSCC).

**Methodology/Principal Findings:**

FOXM1 mRNA and protein expressions were investigated in four independent cohorts (total 75 patients) consisting of normal, premalignant and HNSCC tissues and cells using quantitative PCR (qPCR), expression microarray, immunohistochemistry and immunocytochemistry. Effect of putative oral carcinogens on FOXM1 transcriptional activity was dose-dependently assayed and confirmed using a FOXM1-specific luciferase reporter system, qPCR, immunoblotting and short-hairpin RNA interference. Genome-wide single nucleotide polymorphism (SNP) array was used to ‘trace’ the genomic instability signature pattern in 8 clonal lines of FOXM1-induced malignant human oral keratinocytes. Furthermore, acute FOXM1 upregulation in primary oral keratinocytes directly induced genomic instability. We have shown for the first time that overexpression of FOXM1 precedes HNSCC malignancy. Screening putative carcinogens in human oral keratinocytes surprisingly showed that nicotine, which is not perceived to be a human carcinogen, directly induced FOXM1 mRNA, protein stabilisation and transcriptional activity at concentrations relevant to tobacco chewers. Importantly, nicotine also augmented FOXM1-induced transformation of human oral keratinocytes. A centrosomal protein CEP55 and a DNA helicase/putative stem cell marker HELLS, both located within a consensus loci (10q23), were found to be novel targets of FOXM1 and their expression correlated tightly with HNSCC progression.

**Conclusions/Significance:**

This study cautions the potential co-carcinogenic effect of nicotine in tobacco replacement therapies. We hypothesise that aberrant upregulation of FOXM1 may be inducing genomic instability through a program of malignant transformation involving the activation of CEP55 and HELLS which may facilitate aberrant mitosis and epigenetic modifications. Our finding that FOXM1 is upregulated early during oral cancer progression renders FOXM1 an attractive diagnostic biomarker for early cancer detection and its candidate mechanistic targets, CEP55 and HELLS, as indicators of malignant conversion and progression.

## Introduction

The forkhead box (FOX) protein family of transcription factors exhibit a myriad of biological functions such as regulation of cell cycle, proliferation, apoptosis, differentiation and longevity during embryonic development and adult tissue homeostasis [reviewed in 1]. The FOXM1 transcription factor (previously known as: HFH-11, INS-1, WIN, MPP2/MPHOSPH2 or Trident/FKHL16) has been shown to play important roles in cell cycle progression and mitosis [reviewed in 2]. FOXM1-null mouse embryos were neonatally lethal as a result of the development of polyploid cardiomyocytes and hepatocytes, highlighting the role of FOXM1 in mitotic division [3]. More recently a study using transgenic/knockout mouse embryonic fibroblasts and human osteosarcoma cells (U2OS) has shown that FOXM1 regulates expression of a large array of G2/M-specific genes such as PLK1, NEK2 and CENP-F, and plays an important role in the maintenance of chromosomal segregation and genomic stability [4].

We originally established a link between FOXM1 and tumourigenesis when we demonstrated that FOXM1 was a downstream target of Gli1 in basal cell carcinoma (BCC) and showed that of the three known alternatively spliced isoforms (FOXM1A, B and C), FOXM1B isoform was overexpressed in BCCs [5]. Both transcriptional activators FOXM1B and FOXM1C are upregulated in the majority of solid human cancers [reviewed in 2], [6].

Head and neck squamous cell carcinoma (HNSCC) is the sixth most common cancer worldwide. The average five-year survival of patients is very poor (∼50%) and it has not improved in the last 50 years [reviewed in 7]. This is mainly due to the lack of effective diagnostic and prognostic methods which can guide appropriate treatment strategy at early stages. Treatment often requires extensive maxillofacial surgery which imposes significant morbidity requiring long term multidisciplinary care hence further increasing financial pressures on public health services. Therefore, one of the keys to successful treatment and cure is early detection of tumour formation.

Whilst the FOXM1 gene is now widely accepted to be a commonly upregulated oncogene in majority of human solid cancers, its role and mechanism in cancer initiation and progression remain unclear. Herein, we present that FOXM1 is significantly upregulated in human premalignant and HNSCC tissues and cultured cells. Due to the fact that HNSCC has a strong risk factor association, we questioned the role of FOXM1 in response to common oral carcinogens often present in the oral mucosa. A preliminary screen of oral carcinogens surprisingly showed that nicotine activated endogenous activity of FOXM1 in human oral keratinocytes. As tobacco is one of the main etiological factors for HNSCC and nicotine is one of the major alkaloids found in tobacco, we studied the role of nicotine in carcinogenesis through activation of FOXM1 (isoform B). We now show that FOXM1B is upregulated early in HNSCC development and acts in concert with nicotine to promote anchorage-independent growth. High-density SNP analysis of anchorage-independent transformed cell clones revealed 2 candidate downstream effectors of FOXM1B action, which are overexpressed in malignant SCC lines. The present study investigated the effect of nicotine and the mechanism of FOXM1 in HNSCC using a combination of molecular, cellular, tissue, microarray and bioinformatics approaches.

## Results

### Upregulation of FOXM1 precedes HNSCC malignancy

**Figure 1:**
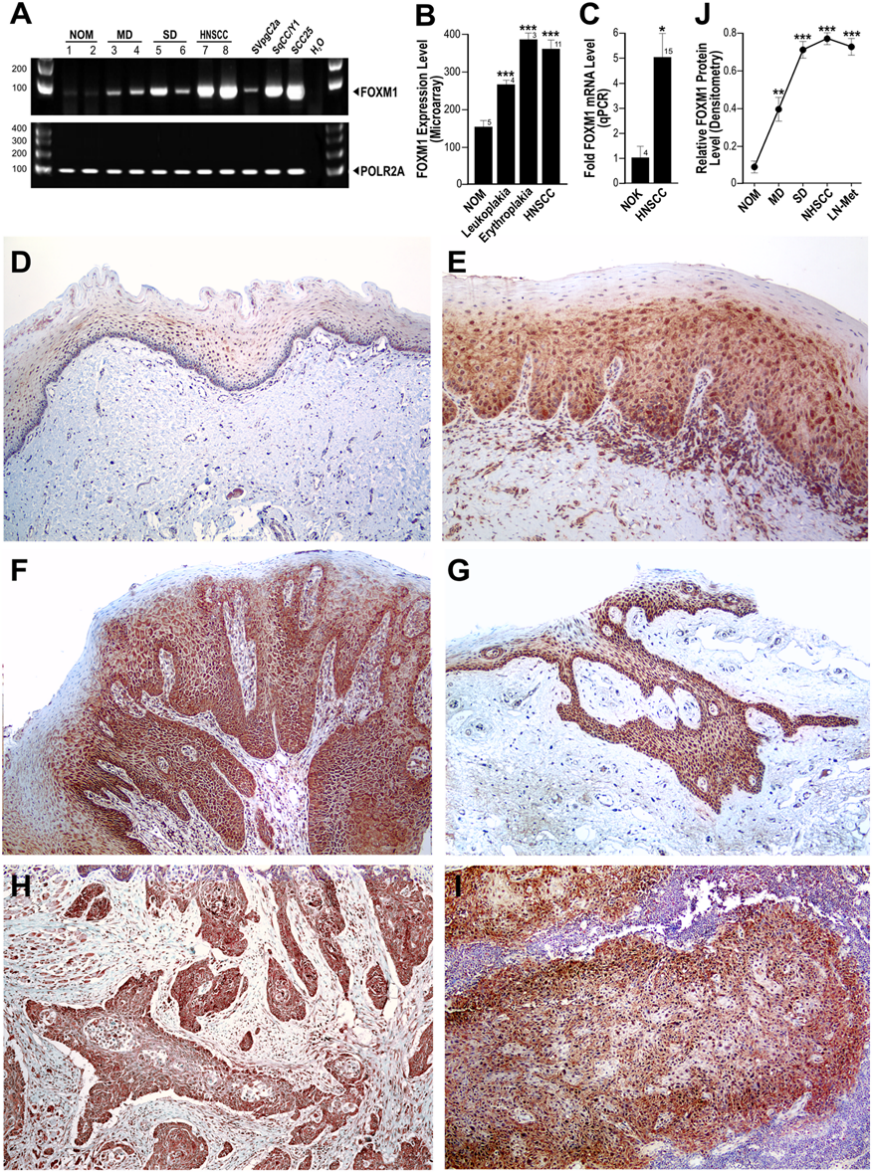
Upregulation of FOXM1 in both human oral premalignant and HNSCC tissues. (A) Semi-quantitative RT-PCR showing the relative expression levels of total FOXM1 mRNA in normal oral mucosa (NOM; #1–2), moderate dysplasia (MD; #3–4), severe dysplasia (SD; #5–6), primary HNSCC (#7–8), premalignant oral keratinocytes SVpgC2a, HNSCC cell lines SqCC/Y1 and SCC25. POLR2A was used as an endogenous reference gene. (B) Bioinformatics analysis of microarray data performed on primary cells extracted from normal oral mucosa (NOM), leukoplakia, erythroplakia and primary HNSCC with sample number as indicated. Statistically significant (***P<0.001) activation of FOXM1 mRNA levels when compared to NOM. (C) Quantitative real-time RT-PCR showing relative fold change in FOXM1 mRNA expression levels in HNSCC-derived keratinocytes (n = 15) compared to normal oral mucosa keratinocytes (NOK; n = 4). (D–I) Immunohistochemistry of FOXM1 protein on a panel of FFPE normal oral mucosa (D), mild/moderate dysplasia (E), moderate/severe dysplasia (F), severe dysplasia/carcinoma in situ (G), primary HNSCC (H) and lymph node HNSCC metastasis (I). (J) Digital pixel densitometry for FOXM1 protein immunoreactivity in a panel of 25 oral tissues (n = 5 in each group) as shown in D–I. **(P<0.01) and ***(P<0.001) indicate statistically significant elevation of FOXM1 protein levels when compared to NOM tissues.

**Figure 2:**
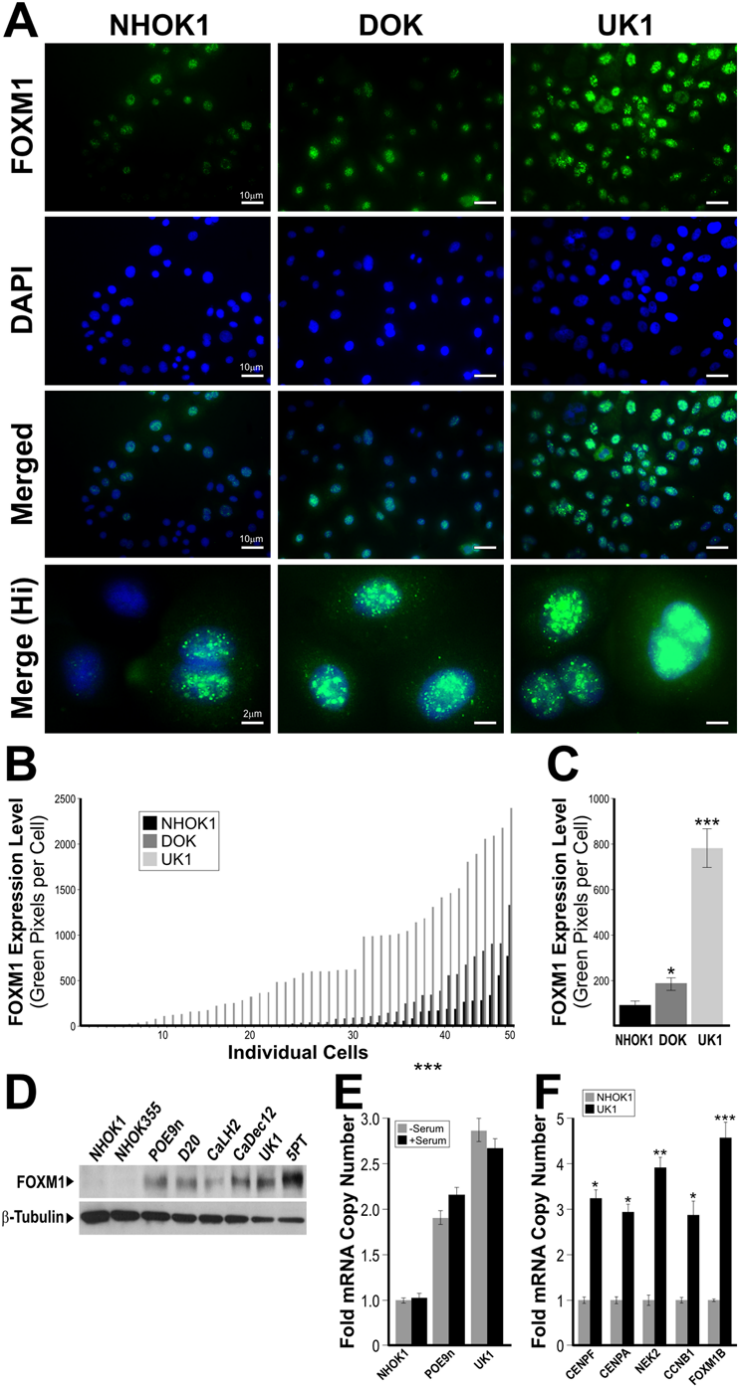
Intracellular expression of endogenous FOXM1 protein in primary cultures of human normal oral mucosa (NHOK1), dysplasia (DOK) and HNSCC (UK1). (A) Fluorescence images showing intracellular localisation of FOXM1 (green) in respective cells counterstained by a nuclear-stain DAPI (blue). The bottom panels are FOXM1 and DAPI merged images of high-power magnification (×400) showing nuclear localisation of FOXM1 protein. (B) Histogram of FOXM1 protein expression levels in 50 cells analysed arranged from low to high FOXM1 levels. (C) Mean FOXM1 protein expression levels in HNOK1, DOK and UK1 cells, n = 50 cells analysed in (B). (D) Immunoblotting for endogenous FOXM1 protein in normal (NHOK1 and NHOK355), dysplasia (POE9n and D20) and HNSCC (CaLH2, CaDec12, UK1 and 5PT) cell lines as indicated. β-Tubulin was immunoblotted for loading control in each sample. (E) Serum starvation (24 h) did not alter the endogenous FOXM1B mRNA levels in primary NHOK1, premalignant POE9n or UK1 HNSCC cells. (F) qPCR data showed that known FOXM1B target genes such as CENPF, CENPA, NEK2 and cyclin B1 are upregulated in UK1 compared to NHOK1 cells. *(P<0.05), **(P<0.01) and ***(P<0.001) indicate significant increase in FOXM1 protein levels when compared to NHOK1 cells.

To investigate the expression levels of endogenous FOXM1 we initially used semi-quantitative RT-PCR on a tissue panel of normal human oral mucosa, oral dysplasia, HNSCC tissues and various oral keratinocyte lines (Fig 1A). Total FOXM1 mRNA expression was low in normal mucosa whilst a trend of progressive increase in expression from premalignant to malignant tissues was detected. In support, a well characterised premalignant buccal keratinocyte line SVpgC2a [8] showed moderate FOXM1 expression, whilst, the malignant HNSCC lines SqCC/Y1 [9] and SCC25 [10] showed significant FOXM1 overexpression. Furthermore, our bioinformatics analysis based on a panel of microarray data [11] showed that FOXM1 mRNA expression was significantly upregulated in both premalignant dysplastic lesions (leukoplakia and erythroplakia) and HNSCC compared to normal oral mucosa (Fig. 1B). In agreement, an independent real-time quantitative RT-PCR (qPCR) study involving 15 patients' HNSCC-derived primary cells showed significant ∼5.1-fold (P<0.001) upregulation of FOXM1 mRNA compared to normal oral keratinocyte samples derived from four different patients (Fig 1C). FOXM1 protein expression was next confirmed by immunohistochemistry on a separate panel of human oral tissues showing significant increase in FOXM1 protein expression in moderate and severely dysplastic lesions, primary HNSCC and lymph node metastasis (Fig. 1D–I). Digital densitometry of these immunostained sections showed a trend of progressive increase in FOXM1 protein levels from moderate dysplasia to lymph node metastasis (Fig. 1J). All control normal oral mucosa (NOM) tissues examined in this study showed low basal levels of FOXM1 mRNA and protein levels. These results are in agreement with a separate experiment using immunofluorescent staining to detect endogenous FOXM1 protein levels on cultured primary normal human oral keratinocyte (NHOK1), dysplastic (DOK) and HNSCC (UK1) cells (Fig. 2A; Supplemental Fig. S1). Digital pixel densitometry was used to quantify the levels of FOXM1 protein expression (Fig. 2B). In NHOK1 cells, FOXM1 protein was detected at low levels mainly within nuclei of dividing cells but not expressed in non-dividing cells. In contrast, FOXM1 expression was highly expressed in both dividing and non-dividing UK1 cells. In DOK and UK1 cells, FOXM1 protein levels were significantly upregulated by 2.4-fold (P<0.05) and 9.2-fold (P<0.001), respectively, over NHOK1 cells (Fig. 2C). Western blotting showed significant upregulation of endogenous FOXM1 protein in both premalignant (POE9n, D20) and HNSCC (CaLH2, CaDec12, UK1, 5PT) cell lines compared to normal oral keratinocytes (NHOK1, NHOK355; Fig 2D). Almost all (n = 6) normal oral keratinocyte patient's samples that we have tested have shown equally low levels of FOXM1 protein (data not shown). Growth factor or serum starvation in an oral premalignant (POE9n) and HNSCC (UK1) cell line did not reduce the endogenous mRNA of FOXM1B in these cells indicating that upregulation of FOXM1 in these cells was not simply due to increased cell proliferation (Fig. 2E). qPCR results showed that a number of known FOXM1B target genes such as CENPF, CENPA, NEK2 and cyclin B1 (CCNB1) were significantly upregulated in UK1 cells compared to NHOK1 (Fig. 2F) showing that FOXM1B in HNSCC cells was transcriptionally active. These results showed for the first time that FOXM1 expression is upregulated early in oral premalignant tissues and its expression persists in both primary HNSCC and lymph node metastasis.

**Figure 3:**
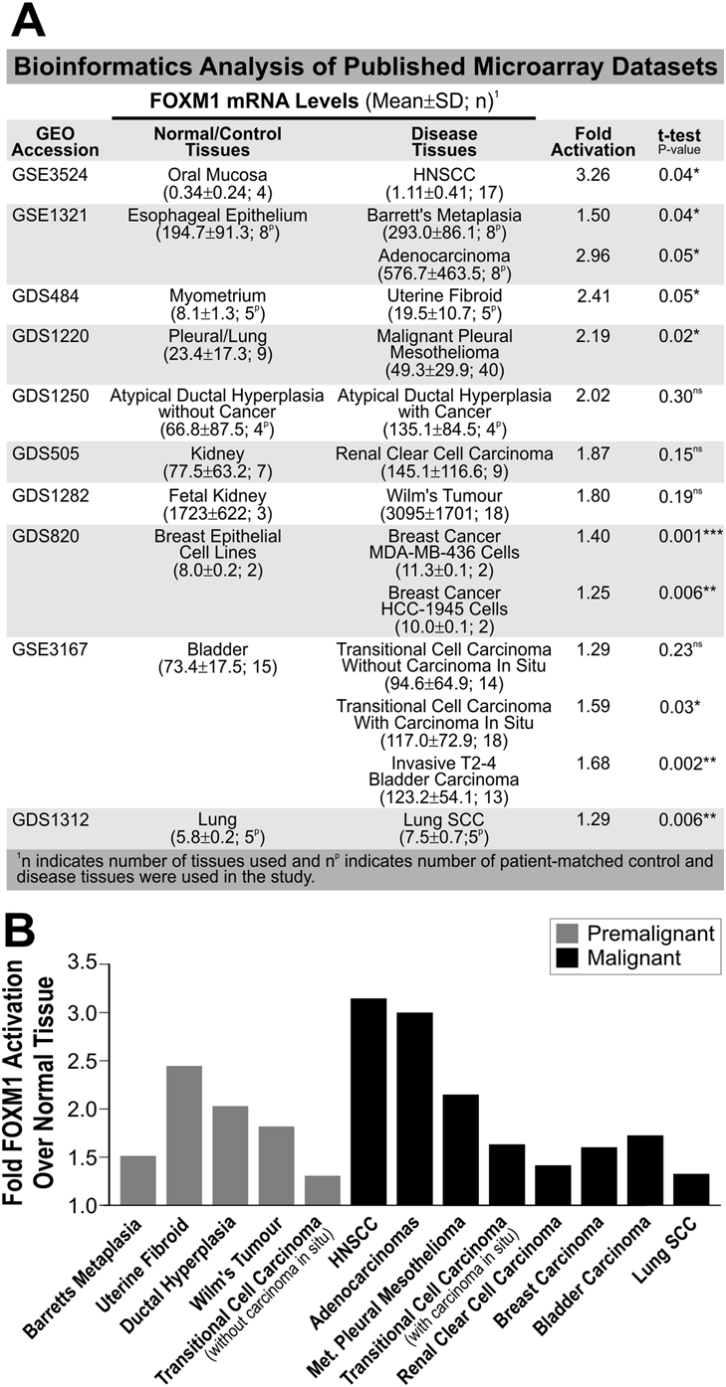
Upregulation of FOXM1 in various human premalignant and primary cancers. (A) Bioinformatics analysis of published microarray data on FOXM1 gene expression levels comparing various human tissue types of normal, premalignant and cancers. (B) Fold activation of FOXM1 gene expression in individual tissue types over normal tissues as indicated.

To further investigate FOXM1 expression in other human tissue types, we performed bioinformatics analyses on published microarray data on premalignant lesions including Barrett's metaplasia, uterine fibroid, atypical ductal hyperplasia that later developed into cancer, all showed statistically significant FOXM1 upregulation (Fig. 3). Analyses of malignant cancer tissues showed that FOXM1 was significantly upregulated in HNSCC (3.1-fold), oesophagus adenocarcinoma (3-fold) and lung malignant mesothelioma (2.4-fold; see Fig. 3B). Taken together, these data confirm that FOXM1 expression is upregulated early in a number of premalignant tissues as well as malignant cancers. This supports a key role for FOXM1 in early oncogenesis.

### Nicotine activates FOXM1 transcriptional activity and promotes FOXM1B-induced anchorage-independent transformation of oral keratinocytes

**Figure 4:**
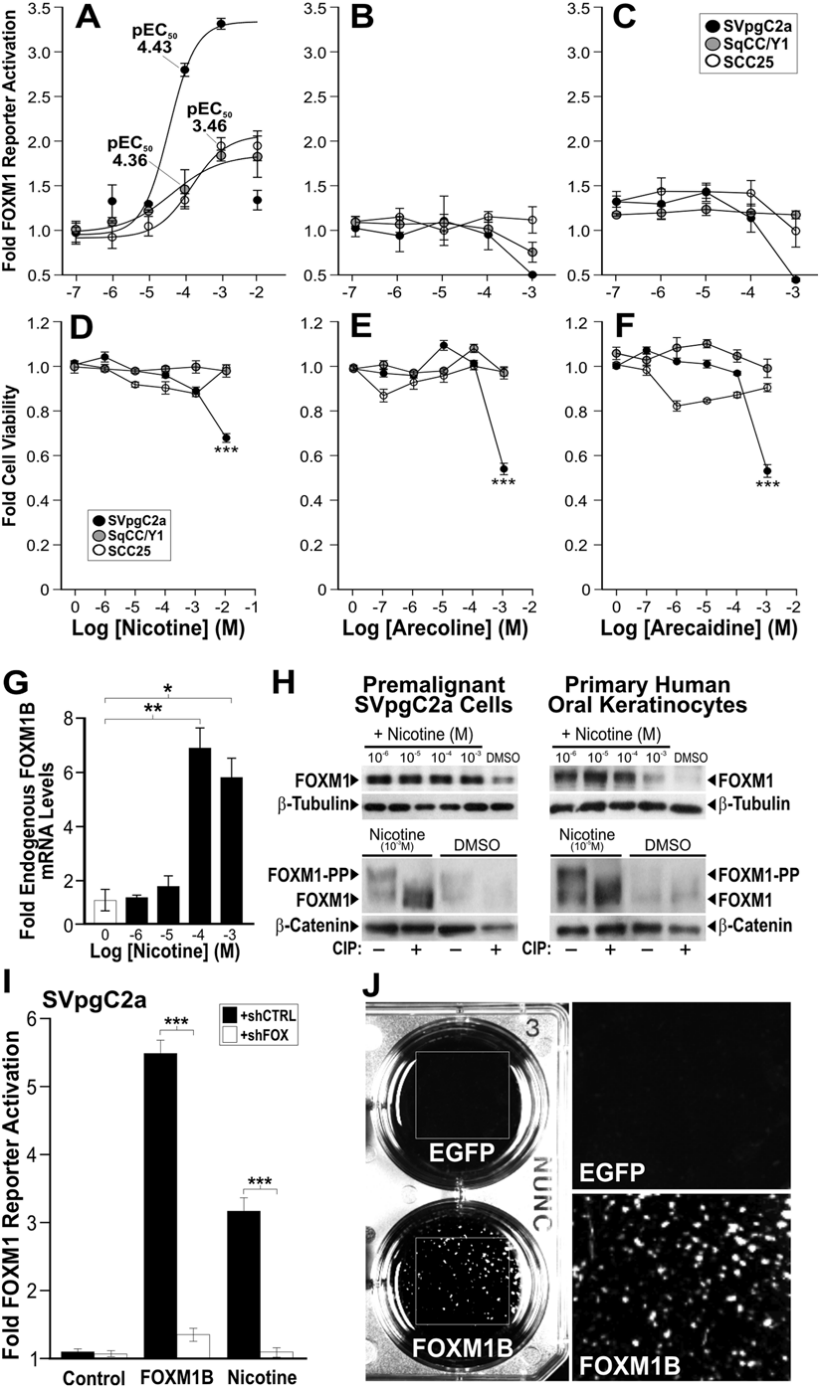
Nicotine activated endogenous FOXM1 transcriptional activity and promoted FOXM1B-induced malignant transformation in human oral keratinocytes. (A–C) Dose-response curves of nicotine (A), arecoline (B) and arecaidine (C) on FOXM1 transcriptional activity in SVpgC2a, SqCC/Y1 and SCC25 cells, respectively. (D–F) Cell viability assays on SVpgC2a, SqCC/Y1 and SCC25 cells treated with the indicated doses of nicotine (D), arecoline (D) and arecaidine (F), respectively. Each data point indicates mean±SEM (n = 3). ***(P<0.001) indicates significant cell death. (G) qPCR shows that nicotine dose-dependently activated endogenous FOXM1B in SVpgC2a cells. (H) Immunoblotting shows that nicotine dose-dependently activated endogenous FOXM1 protein in both the SVpgC2a (left panel) and primary normal human oral keratinocytes (right panel) as indicated. Bottom panels showed that protein lysates were either pretreated with or without calf-intestinal phosphatase (CIP) prior to immunoblotting. Nicotine at respective optimal doses for each cell type showed reduction of the phosphorylated FOXM1 (FOXM1-PP, top bands) in CIP-treated lysates. β-Tubulin and β-catenin were used as loading control markers. (I) Reversal of nicotine-induced FOXM1 activation by RNA interference shFOX. FOXM1 transcriptional activity was assayed in SVpgC2a cells co-transfected with either shCTRL (control shRNA; solid bars) or FOXM1-specific shFOX (open bars) in either control (mock transfection), FOXM1-overexpressed or nicotine (1 mM)-treated cells, as indicated. ***(P<0.001) indicates significant repression of FOXM1 transcriptional activity over control cells. (J) Anchorage-independent cell transformation assays of EGFP or FOXM1B-overexpressing and nicotine-treated (10 mM) SVpgC2a cells.

Tobacco and betel quid are two known risk factors for the development of HNSCC. In order to investigate the role of FOXM1 in response to these oral risk factors, we tested our hypothesis that nicotine (one of the major alkaloids found in tobacco), arecoline and arecaidine (the main alkaloids found in areca nuts in betel quid) can directly activate endogenous FOXM1 transcriptional activity in cells. A FOXM1 luciferase reporter assay was performed on a premalignant buccal keratinocyte line SVpgC2a [8], a malignant human buccal squamous cell carcinoma line SqCC/Y1 [9] and an tongue HNSCC line SCC25 [10], [12]. Nicotine, at physiologically relevant doses for tobacco chewers, was found to induce a dose-dependent activation of endogenous FOXM1 activity in all three cell types (Fig. 4A). The effect of nicotine on FOXM1 activity was more potent on the premalignant SVpgC2a cells (pEC_50_ 4.43) than on the two malignant lines SCC25 (pEC_50_ 3.46) and SqCC/Y1 (pEC_50_ 4.36). In addition, nicotine was able to produce a higher level of FOXM1 activity in SVpgC2a (>3-fold) cells compared to SqCC/Y1 or SCC25 (<2-fold). This could be due to the higher endogenous FOXM1 levels already present in SqCC/Y1 and SCC25 (see Fig. 1A). Arecoline and arecaidine showed no effect on FOXM1 transcriptional activity in either cell type (Fig. 4B–C). None of these alkaloids at their respective highest doses affected SCC25 cell viability (Fig. 4D–F). However, in the case of SVpgC2a cells, the highest dose of nicotine (10^−2^ M), arecoline (10^−3^ M) and arecaidine (10^−3^ M) showed significant decrease in cell viability (Fig. 4D–F) indicating that the oral premalignant cell line is generally more sensitive compared to the two HNSCC cell lines. In line with the pattern of sensitivity, nicotine greater than 10^−3^ M (1 mM) were toxic to primary normal human oral keratinocytes (data not shown). Endogenous mRNA levels of FOXM1B were found to be dose-dependently elevated by nicotine (Fig. 4G). Consistently, FOXM1 protein levels were also dose-dependently increased by nicotine in both the premalignant SVpgC2a cell line and in normal primary oral keratinocytes (Fig. 4H). Furthermore, calf-intestinal alkaline phosphatase (CIP; Fig. 4H lower panels) treatment in protein lysates from either control DMSO or nicotine treated cells showed that FOXM1 protein becomes heavily phosphorylated after nicotine treatment, which is a well established mechanism leading to FOXM1 protein stabilisation and increased transactivation [13], [14]. Using a FOXM1 specific short-hairpin RNA interference plasmid (shFOXM1) to knockdown endogenous FOXM1 gene expression, nicotine-induced FOXM1 activity was significantly inhibited confirming the direct effect of nicotine on FOXM1 activation. The control shCTRL bearing a random sequence did not inhibit nicotine-induced FOXM1 activity (Fig. 4I).

Given that SVpgC2a cells were more sensitive to nicotine-induced cell death than SqCC/Y1 or SCC25 cells, we hypothesized that upregulation of the cancer-associated FOXM1B isoform in SVpgC2a may protect cells from nicotine toxicity relevant to direct nicotine exposure in tobacco chewers. To test this, we compared nicotine toxicity in SVpgC2a (bearing relatively lower levels of endogenous FOXM1B; see Fig. 1A) with SqCC/Y1 and SCC25 (both bearing high endogenous FOXM1B levels; see Fig. 1A). FOXM1B, overexpression in SVpgC2a but not the empty vector significantly (P<0.001) protected cells from nicotine-induced cell death (Supplemental Fig. S2). Whereas nicotine did not induce cell death in SqCC/Y1 and SCC25 cells. We subsequently hypothesized that upregulation of FOXM1B in SVpgC2a may help these cells to gain survival advantage and may promote anchorage-independent transformation following nicotine exposure. Indeed, we have found that upregulation of FOXM1B induces anchorage-independent growth and malignant transformation in SVpgC2a following nicotine exposure (Fig. 4I). FOXM1B overexpression alone in SVpgC2a cells did not produce any transformed clones in the soft-agar colony forming assay (data not shown). Control EGFP-expressing SVpgC2a cells exposed to nicotine did not produce transform colonies (Fig. 4J). Collectively, our results showed that upregulation of FOXM1B protected cells from nicotine-induced toxicity and promoted anchorage-independent growth. Although many HNSCC lines do not grow well in agar, some do and most grow better than normal keratinocytes [12], suggesting that anchorage independence is a valid assay of malignant transformation.

### FOXM1B induces non-random genomic instability

**Figure 5:**
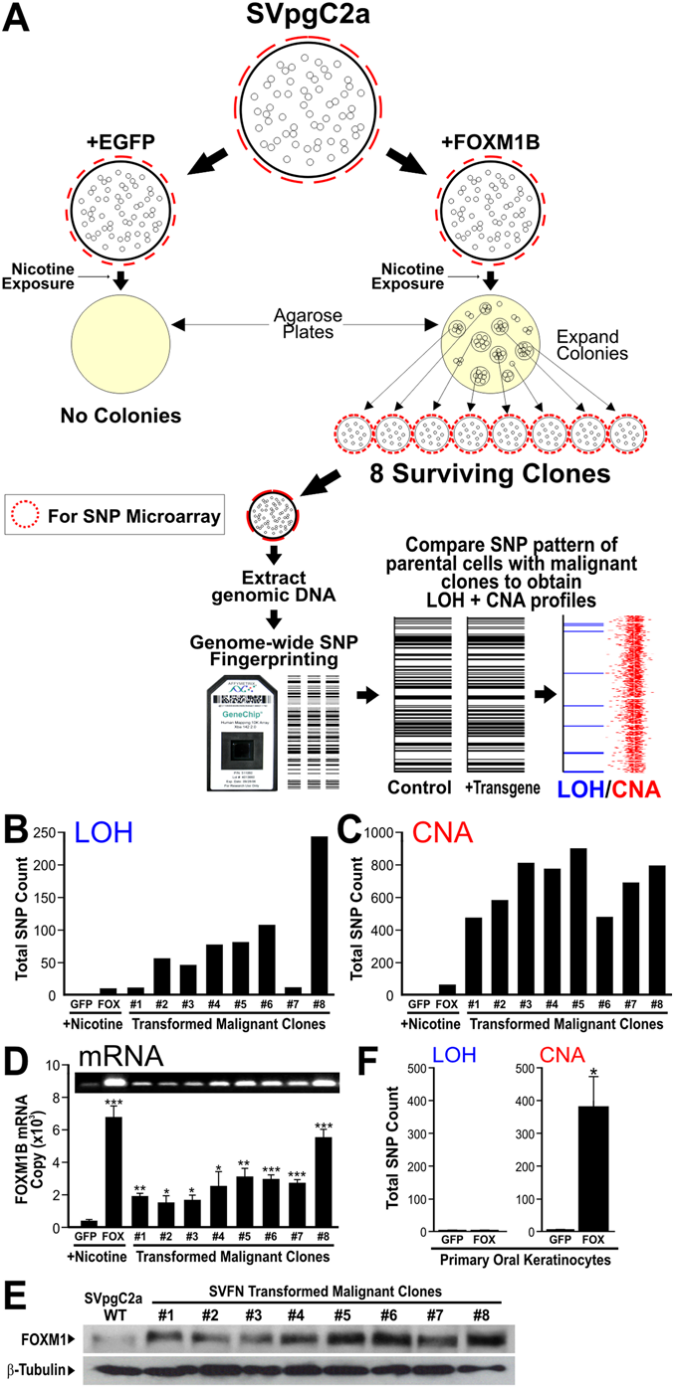
Upregulation of FOXM1B induces non-random genomic instability. (A) A schematic flow diagram showing the approach used in this study to systematically ‘trace’ the genomic instability patterns (LOH and CNA) within the genomes of 8 individual nicotine/FOXM1B-transformed clones using SNP microarrays. Levels of LOH (B), CNA (C), FOXM1B mRNA (using isoform-specific qPCR, see Supplemental Fig. S3) (D) and FOXM1 protein (E) in respective cell clones. (F) Levels of LOH and CNA in primary human normal oral keratinocytes expressing either EGFP or FOXM1B (n = 3). *(P<0.05), **(P<0.01) and ***(P<0.001) indicate significant increase over control cells.

With an aim to identify FOXM1B-target genes involved in malignant transformation, we next questioned if the FOXM1B-transformed clones we generated in Fig. 4H may harbour ‘signature’ genomic instability loci associated with malignant transformation. Eight transformed clones (SVFN1-8) were isolated from the soft-agar plates and grown into individual cell lines and their genomic DNA extracted for genome-wide SNP array profiling (Fig. 5A) to obtain genomic instability in the form of loss of heterozygosity (LOH) and copy number abnormality (CNA). LOH and CNA loci were identified by comparing SNP genotype profiles between test cell samples with wild-type parental SVpgC2a cells (non-transformed). Nicotine treatment alone did not result in genomic instability in the wild-type parental SVpgC2a cells (data not shown) or in the non-transformed SVpgC2a cells expressing EGFP and treated with nicotine (Fig. 5B–C). Non-transformed nicotine-treated FOXM1B expressing cells showed low levels of LOH and CNA (loss) in chromosome 13 (Supplemental Fig. S3). Whereas, all 8 transformed SVFN clones showed significantly elevated LOH and CNA. All 8 SVFN clones had significantly higher levels of FOXM1B mRNA (Fig. 5D) indicating that these clones were derived from FOXM1B-transduced parental cells. Using FOXM1 isoform-specific qPCR (Supplemental Fig. S4), FOXM1A and FOXM1C mRNA were not differentially expressed between the wild-type SVpgC2a and SVFN cells (data not shown). In agreement with the mRNA levels (Fig. 5D), western blotting showed increased FOXM1 protein levels in all the 8 SVFN clones compared to wildtype SVpgC2a cells confirming that FOXM1 expression is retained and that upregulation of FOXM1B confers a selective advantage required for anchorage-independent transformation (Fig. 5E). SNP analysis on primary human oral keratinocytes showed that whilst FOXM1B overexpression alone was not sufficient to induce LOH, we observed significant (Fig. 5F) elevation of CNA indicating that CNA precedes LOH. This is in agreement with the higher frequency of CNA than LOH within the SVFN clones (compare Fig. 5B and 5C).

### FOXM1 target genes identification within recurrent LOH loci

**Figure 6:**
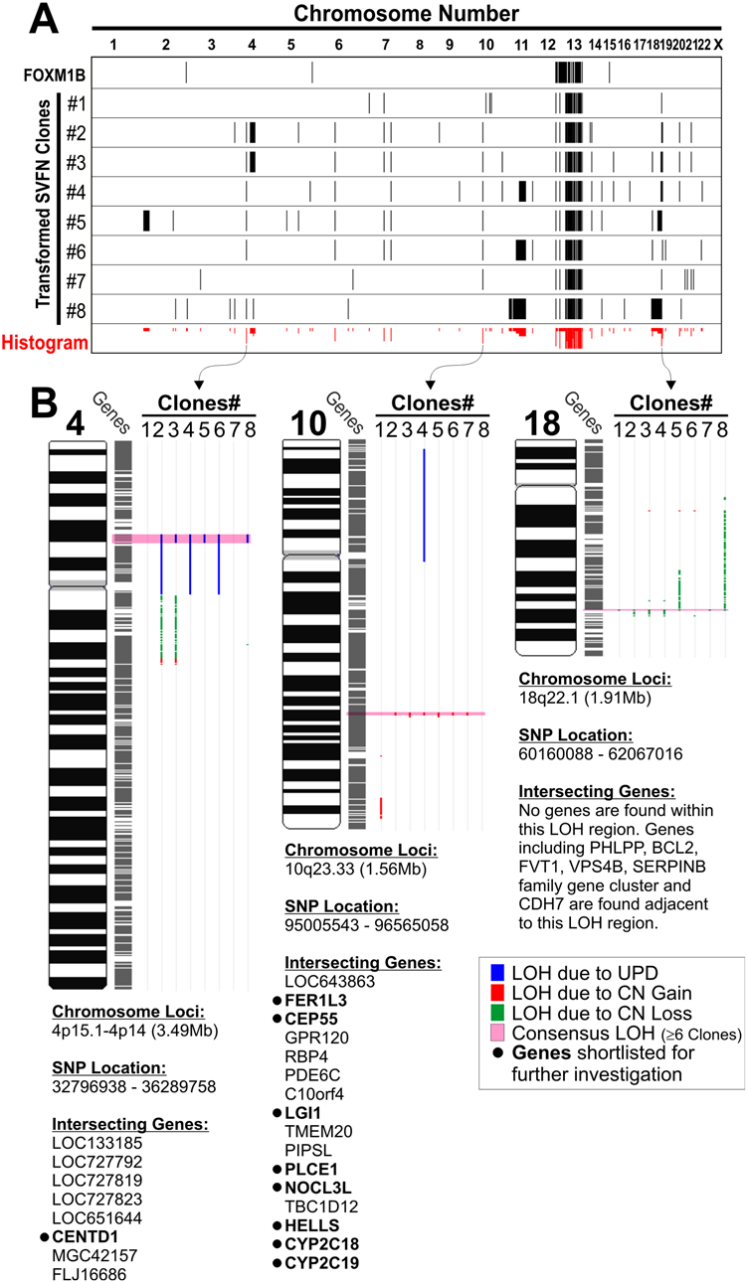
Identification of consensus LOH micro-loci in FOXM1B-transformed SVpgC2a cells. (A) Alignment of genome-wide LOH profiles of non-transformed (FOXM1B) and FOXM1B-transformed SVpgC2a clones (SVFN1-8). Black lines indicate LOH at a given SNP loci. Histogram (red bars) displays consensus regions of LOH across the 8 SVFN transformed clones. (B) Magnified and detail chromosomal view of the three consensus micro-loci within chromosome 4, 10 and 18 with a list of genes located within each locus as indicated. Uniparental disomy (UPD, blue); copy number gain (CN gain, red); copy number loss (CN loss, green); consensus LOH loci in at least 6 SVFN clones (pink).

**Figure 7:**
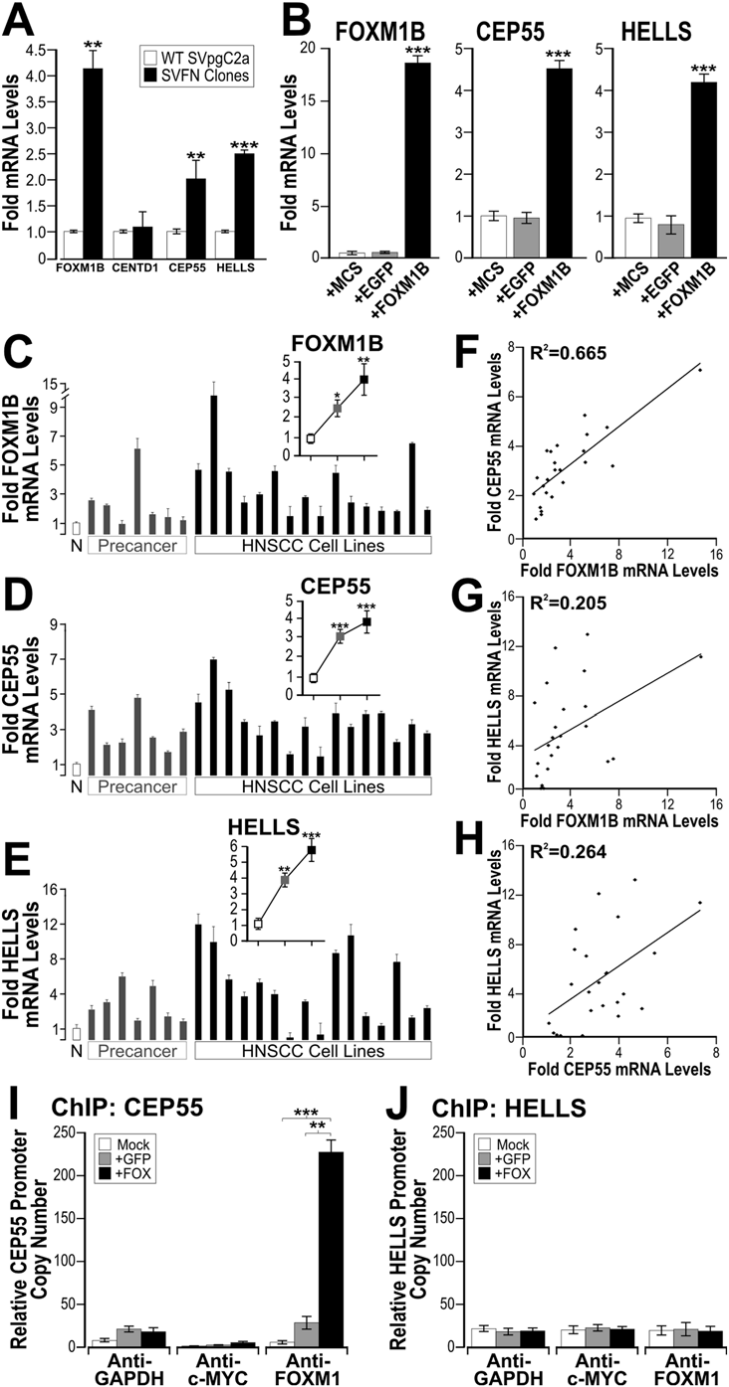
CEP55 and HELLS are putative FOXM1B target genes and are upregulated in both premalignant and HNSCC cell lines. (A) mRNA levels of CEP55 and HELLS but not CENTD1 were significantly upregulated in SVFN transformed clones (solid bars) compared to non-transformed SVpgC2a parental wild-type cells (open bars). (B) Exogenous overexpression of FOXM1B (determined using isoform-specific qPCR, see Supplemental Fig S3), but not MCS (empty vector) or EGFP, in primary human normal oral keratinocytes (NHOK1) showed significant (***P<0.001) induction of endogenous mRNA of CEP55 and HELLS, respectively. Fold mRNA expression levels of FOXM1B (C), CEP55 (D) and HELLS (E) in a panel of oral premalignant cell lines (SVpgC2a, DOK, D19, D20, POE9n-hTERT, POE9n and OKT6) and HNSCC (CA1, UK1, CaLH2, CaLH3, CaDec11, CaDec12, H357, 5PT, PE3/JA, VB6, CaLH2-R, BICR31, SCC4, SCC9, SCC15 and SqCC/Y1) compared to the mean±SEM (n = 8) of normal primary human oral mucosa cells (NHOK1-5, 16, 376, 881). Insets show mean±SEM fold mRNA expression levels of normal (n = 8), oral premalignant (n = 7) and HNSCC cells (n = 16). All three genes show statistically significant (*P<0.05; **P<0.01; ***P<0.001) upregulation in both oral premalignant and HNSCC cell lines. (F) Linear regression analysis between FOXM1B and CEP55 expression in the panel of cell lines used in C and D. (G) Linear regression analysis between FOXM1B and HELLS expression in the panel of cell lines used in C and E. (H) Linear regression analysis between CEP55 and HELLS expression in the panel of cell lines used in D and E. Degree of correlation values (R^2^) are given in each panel as indicated. (I–J) ChIP-qPCR assays for promoters of CEP55 and HELLS in normal keratinocytes transduced with either Mock (empty virus), EGFP or FOXM1B. Antibodies for GAPDH and cMYC were used as controls for immunoprecipitiation. Representative qPCR curves and melting peaks of each ChIP fractions could be found in Supplemental Fig. S7.

Comparison of genome-wide LOH profiles of the 8 SVFN transformed clones revealed a non-random pattern of genomic instability induced by FOXM1B and nicotine (Fig. 6A). The chromosome 13 LOH retained in all the 8 SVFN transformed clones confirms that the clones originated from FOXM1B overexpressed cells. Apart from chromosome 13, the most common recurrent LOH loci (>6 clones) detected in the 8 clones were found in chromosome 4, 10 and 18 (Fig. 6B). Using a selection criterion based on putative functional relevance to oncogenesis, 9 candidate FOXM1B-induced transformation target genes found within these common loci were short listed for further investigation. We first screened gene expression levels using qPCR comparing non-transformed SVpgC2a cells and the SVFN transformed clones to identify putative FOXM1B-target genes. Of the 9 shortlisted genes, CENTD1, CEP55 and HELLS, showed a correlation between mRNA levels and their respective copy number status (Fig. 7A). CENTD1 was found to be located within a locus of uniparental disomy (UPD) with no change in copy number and consistently qPCR data showed no change in CENTD1 mRNA levels in the SVFN clones and as a result this gene was not investigated further. Whereas, CEP55 and HELLS located within a copy number gain loci showed significantly elevated levels of mRNA in the SVFN clones. This suggests that CEP55 and HELLS may be targets of FOXM1B for malignant transformation.

### CEP55 and HELLS are downstream targets of FOXM1

To investigate if FOXM1B may be driving the expression of CEP55 or HELLS genes in oral keratinocytes, we transduced primary human oral keratinocytes with retrovirus bearing either MCS (empty viral vector), EGFP or FOXM1B and subsequently mRNA was harvested from these cells for qPCR. Indeed, qPCR data showed that upregulation of FOXM1B in primary oral keratinocytes led to significant activation of CEP55 (4.43-fold) and HELLS expression (4.21-fold; Fig. 7B). Consistent with the notion that CEP55 is a target of FOXM1B, our bioinformatics analysis of published microarray data (GEO Accession: GDS1477) in FOXM1B-knockdown (by siRNA) breast cancer cells [15] showed significant downregulation of CEP55 expression (Supplemental Fig. S5). Similar bioinformatics analysis could not be performed for HELLS because no data was available.

We next investigated the gene expression correlation of FOXM1B with CEP55 and HELLS using qPCR in a panel of 8 normal human oral keratinocytes, 7 oral premalignant and 16 HNSCC cell lines (Fig 7C–H). As before, the endogenous levels of FOXM1 protein *in vivo* (Fig. 1D–J), and *in vitro* (Fig. 2, Fig. S1) and the endogenous FOXM1B mRNA *in vitro*, were significantly upregulated in premalignant and HNSCC cells (Fig. 7C). Endogenous mRNA levels of CEP55 (Fig. 7D) and HELLS (Fig. 7E) were both significantly upregulated in oral premalignant and HNSCC cell lines. Linear regression analyses showed that mRNA expression profile of CEP55 (Fig. 7F; R^2^ = 0.665) correlated well with FOXM1B expression profile across the panel of oral premalignant and HNSCC cell lines. HELLS mRNA expression profile showed a weaker correlation with either FOXM1B (Fig. 7G; R2 = 0.205) or CEP55 (Fig. 7H; R^2^ = 0.264). These results are in completely agreement with the bioinformatics analysis of published microarray data (GEO Accession: GDS1548; Supplemental Fig. S6) on a panel of 4 normal and 16 HNSCC patients' tissues whereby FOXM1 (Fig. S6A), CEP55 (Fig. S6B) and HELLS (Fig. S6C) are all upregulated in HNSCC and that CEP55 (R^2^ = 0.80; Fig. S6D) correlated better than HELLS (R2 = 0.61; Fig. S6E) with FOXM1 expression.

### CEP55 is a direct transcriptional target of FOXM1

We next investigated whether CEP55 and HELLS are direct transcriptional targets of FOXM1 using chromatin immunoprecipitation qPCR (ChIP-qPCR) in normal human keratinocytes. ChIP-qPCR results for the anti-FOXM1 (but not anti-GAPDH or anti-cMYC) ChIP fraction showed that promoter copy number of CEP55 were significantly higher in FOXM1B-transduced cells compared to either mock or EGFP-expressing cells (Fig. 7I and Supplemental Fig. S7A). However, promoter copy number of HELLS was insignificant (Fig. 7J and Supplemental Fig. S7B) in any of the IP fractions, suggesting that CEP55, but not HELLS, is a direct target of FOXM1. Neither anti-GAPDH nor anti-cMYC showed significant levels of either CEP55 or HELLS promoter fragments indicating that the assay is specific. To further validate these ChIP fractions, known FOXM1 transcriptional targets such as cyclin B1 [16] (Supplemental Fig. S8A) and VEGF [17] (Supplemental Fig. S8B–C) showed significantly higher copy numbers of respective promoters in the anti-FOXM1 ChIP fractions. FOXM1 did not bind to the promoter of the tumour suppressor p16 (CDKN2A; Fig. S8D).

## Discussion

The FOXM1 transcription factor is commonly overexpressed in a variety of human malignancies and several studies have suggested that this may be associated with malignant transformation [reviewed in 2], [6]. Despite the fact that the FOXM1 gene locus (12p13.3) has been reported to be amplified in HNSCC [18], its mechanism of action in oral carcinogenesis has not yet been investigated. This study provided the first evidence that FOXM1 is upregulated in oral dysplasias and HNSCC samples collected from various geographically distinct patient cohorts consisting of both Caucasians and Asians (Fig. 1,2). Our bioinformatics analysis on published microarray data on various other types of human premalignant lesions and primary cancers confirmed that FOXM1 gene is commonly upregulated in a number of premalignant tissues supporting its ubiquitous role in early oncogenesis.

**Figure 8:**
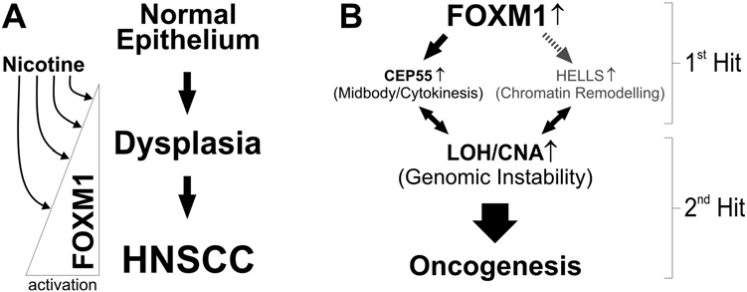
A model mechanism of FOXM1-induced oncogenesis. (A) FOXM1 expression is upregulated early during the progression from dysplasia to HNSCC malignancy. Nicotine may promote tumour development by synergising with the upregulation of FOXM1 during oncogenesis. (B) Oncogenic 1^st^ hit represents overexpression of FOXM1 which directly activates CEP55 but indirectly activates HELLS expressions. This may destabilise the genome through aberrant mitotic division/cytokinesis and/or epigenetic modification events induced by CEP55 and HELLS, respectively. Oncogenic 2^nd^ hit represents the subsequent accumulation of further genomic instability (for example 10q23 amplification) which promotes oncogenesis.

Tobacco (both smoke and smokeless forms) and betel quid use are the main risk factors for the development of HNSCC [19]. At concentrations (0.01–10 mM) relevant to tobacco chewers, nicotine, but not arecoline and arecaidine was found to activate endogenous FOXM1 transcriptional activity through upregulation of mRNA, protein levels and increased protein stabilisation through phosphorylation in a dose-dependent fashion (Fig. 4). The co-carcinogenic effect of nicotine supports the finding that the risk of developing HNSCC is increased to 15 times higher in patients chewing betel quid with tobacco compared to chewing betel quid alone [20]. Nicotine is currently perceived as a safe and non-carcinogenic substance often used in tobacco replacement therapy, despite increasing evidence that nicotine exhibit anti-apoptotic effect in oral epithelial cells [21], [22]. The growth promoting effect of nicotine may explain the effect of nicotine on FOXM1 activity. It has been shown that salivary nicotine concentration can reach millimolar concentrations in tobacco chewers. Nicotine chewing gum (eg. Nicorette, Nicotinell) contains between 2–4 mg nicotine and is expected to produce millimolar concentrations in the oral cavity. Some nicotine skin patches contain up to 21 mg nicotine (NicoDerm) which can deliver up to ∼3 mM/hr (500 ng.hr/ml) of plasma nicotine level [23] and is expected to produce much higher nicotine concentration locally on the skin. Although no evidence to date links nicotine exposure alone to human cancer, it has been previously shown that nicotine is co-carcinogenic with a tobacco smoke derivative benzo(a)pyrene in causing skin cancer in mice [24]. A recently marketed nicotine-supplemented drink (NICLite®, Nichonica Ltd., California, USA) contains 4 mg nicotine (2 cigarettes worth) and will produce up to 0.8 mM (125 ng/ml) plasma nicotine level within 6 hrs. We have now shown that a concentration of nicotine as low as 0.1 µM was able to elevate FOXM1 protein levels in primary human oral keratinocytes (Fig. 4H). More alarmingly, nicotine-laced alcoholic drinks (eg. Nicotini, a tobacco infused vodka or martini) are being offered in bars and restaurants following smoking bans in various developed countries. It has been reported that the synergistic actions of severe alcohol and tobacco abuse can have over 100-fold greater risk of developing HNSCC [25]. Our findings that nicotine can directly activate the FOXM1 oncogene and the combined action of FOXM1B upregulation and nicotine exposure may induce malignant transformation (Fig. 8A) should prompt a reassessment of the widespread use of nicotine-based tobacco replacement therapies.

We believe that FOXM1B overexpression (1^st^ hit) alone in the premalignant SVpgC2a cells may eventually induce transformation if cells were subjected to prolonged cell culture to allow sufficient time to accumulate further oncogenic hits. We did not see spontaneous FOXM1B-induced transformation during the course of our study (6 months; data not shown). Hence, a semi-toxic dose of nicotine (10 mM – a concentration relevant to tobacco chewers) was used to expedite the selection of FOXM1B-transformed SVpgC2a cells. Whilst nicotine at lower doses (0.1–1 mM) activated FOXM1, nicotine at 10 mM did not activate FOXM1 activity due to toxicity (Fig. 4A). This dose-related dual effect of nicotine may explain its co-carcinogenic activity, ie, at low doses it activates FOXM1B and at high doses it induces cell death thereby enhancing oncogenic selection. Because FOXM1B provides survival advantage, we believe that the fluctuating nicotine exposure in long-term tobacco users serves as a selection mechanism for oral cells with increased FOXM1B expression thereby increasing the risk of malignant transformation through subsequent genomic instability and accumulation of further oncogenic hits.

We have shown for the first time using genome-wide SNP profiling approach that acute upregulation of FOXM1B alone in primary normal human oral keratinocytes was able to induce random genomic instability in the form of CNA but not LOH (Fig. 5F), indicating that FOXM1 overexpression alone is not sufficient to induce LOH formation in normal cells. Nevertheless, the upregulation of FOXM1B alone in a premalignant oral keratinocyte line was able to induce LOH involving the whole of chromosome 13. This is consistent with our previous finding that chromosome 13 (13q14–q33) was a hotspot LOH in a premalignant condition oral submucous fibrosis [26] and others have shown that LOH in 13q21 is linked to the progression of HNSCC [27]. The nicotine/FOXM1B-transformed malignant cell clones (SVFN1-8; Fig. 5 & 6) exhibited multiple additional LOH/CNA loci. The non-random pattern of LOH/CNA loci in these malignant clones supports the phenomenon of field cancerisation often seen in HNSCC [19], [28] and numerous other cancers such as lung, oesophagus, vulva, cervix, colon, breast, bladder and skin [29].

Although FOXM1B at physiological levels has been reported as a regulator of DNA repair [30], its upregulation is likely to interfere with the normal DNA repair mechanism leading to enhanced genomic instability rather than enhanced DNA repair. It is known that levels of many cell cycle-related genes are tightly regulated and any deviations from their physiological critical concentrations may cause problems. It has been shown that silencing FOXM1 leads to genomic instability via aberrant expression of mitotic spindle assembly genes such as CEPN-F, Aurora B and Plk1, which are putative targets of FOXM1 [4]. We have shown that overexpression of FOXM1B can also cause genomic instability. In support, numerous studies have demonstrated that proteins which are important in mitotic division are often found amplified in human cancers giving rise to genomic instability [31]. Whilst DNA repair and genomic instability may, or may not, be directly inter-linked, FOXM1B induction may trigger both DNA repair (aberrant repair) as well as genomic instability. It is important to emphasise that our data (Fig. 1B) showed that FOXM1 gene is significantly upregulated in genomically unstable oral premalignant (dysplasia and erythroplakia) and HNSCC samples [11], [32]. Moreover, upregulation of FOXM1B in a wide variety of human cancers [6] is consistent with our data that upregulation of FOXM1B causes genomic instability. The findings that FOXM1B induces DNA repair genes such as XRCC1 and BRCA2 [30] and the fact that most cancers contain genomic instability [33] suggest that activation of DNA repair and genomic instability may not be mutually exclusive in cancer cells. Furthermore, the upregulation of FOXM1B in majority of human cancers suggests that gain of FOXM1B function is an important step in oncogenesis.

In search of FOXM1B-targeted malignancy associated genes, micro-regions of consensus LOH loci shared amongst the 8 malignant clones were compared. The most common recurrent LOH loci were found in 4p, 10q and 18q. Whilst this study did not identify candidate genes within the 4p and 18q, these two loci have been previously linked to HNSCC progression [34]. Our previous study showed that LOH in 10q22–24 and 18q21 were hotspots in oral premalignancy [26] and others have linked 10q with poor HNSCC prognosis [35] and 18q with metastasis of HNSCC [36]. Within the amplified 10q23.33 LOH locus, we have identified a centrosomal protein CEP55 (also known as URCC6, C10orf3 or FLJ10540) and a stem cell marker/DNA helicase/chromatin remodelling factor HELLS (also known as LSH, PASG, SMARCA6, FLJ10339 or Nbla10143) [37] to be likely mediators of FOXM1B-induced malignant transformation. CEP55 and HELLS gene amplifications were found to be the commonest among the transformed keratinocyte clones. In support, the endogenous mRNA of both CEP55 and HELLS were significantly activated by constitutive expression of FOXM1B in primary human normal oral keratinocytes. Consistently, bioinformatics analysis of published microarray data in FOXM1B-knockdown (by siRNA) breast cancer cells [15] showed significantly decreased levels of CEP55 (Supplemental Fig. S5). Our ChIP-qPCR results suggest that FOXM1 protein binds directly to the promoter of CEP55 (Fig. 7I) but not HELLS (Fig. 7J) indicating that CEP55 is a direct transcriptional target of FOXM1, whilst HELLS may be an indirect target. This finding may explain the lower correlation between HELLS and FOXM1 expression (Fig. 7G and Supplemental Fig. S6E).

The upregulation of CEP55 and HELLS by FOXM1B in the absence of nicotine suggests that nicotine acts as an upstream enhancer of FOXM1B activity. Nicotine alone may not be sufficient to achieve a critical FOXM1B threshold level required for malignant transformation. Consistent with this notion, nicotine alone failed to upregulate CEP55 or HELLS mRNA expression (data not shown). This implies that nicotine exerts its co-carcinogenic properties primarily through the enhancement of FOXM1B rather than direct activation of CEP55 or HELLS. Since FOXM1B alone can activate *de novo* expression of CEP55 and HELLS, we hypothesised that this *de novo* activation constitute an initial oncogenic stress (1^st^ hit) which subsequently led to a secondary genomic instability event resulting in a sustained CEP55 and HELLS expression through copy number amplification of 10q23 loci (2^nd^ hit). According to this model (Fig. 8B), at later stages of malignancy, the expression of CEP55 and HELLS may or may not be dependent on FOXM1B. In support for this model, it has been shown previously [38] that upregulation of cyclin D1 (CCND1) expression was detected in oral hyperplasia/premalignant lesions and CCND1 gene locus (11q13) amplification was found in early dysplasia, carcinoma in situ and also metastatic HNSCC. Furthermore, upregulation of CCND1 gene expression was detected in premalignant lesions adjacent to HNSCC tumours with amplified gene locus. In contrast no upregulation of CCND1 gene expression was detected in the premalignant lesions adjacent to non-amplified tumours. Based on this model, we therefore hypothesised that upregulation of CEP55 and HELLS precedes gene amplification during malignant transformation induced by FOXM1B.

Both CEP55 and HELLS mRNA levels were found to be significantly elevated in both oral premalignant and HNSCC cell lines compared to normal oral keratinocytes (Fig. 7D–E). Consistent with their roles in malignancy, CEP55 is upregulated in hepatocellular carcinoma and its overexpression induces anchorage-independent growth, enhanced cell growth at low serum levels and induction of tumourigenesis in nude mice [39]. FOXM1 is known to be overexpressed in hepatocellular carcinoma [40]. A recent study involving 3 independent breast cancer microarray datasets containing a total of 699 patients revealed that CEP55 and FOXM1 are amongst the signature prognostic markers which predicts breast cancer outcome [41]. HELLS has been implicated in leukaemia [42], non-small cell lung cancer [43], breast cancer [44], and melanoma [45]. HELLS was recently identified as one of the consensus genes expressed in human embryonic stem cells [37]. The role of CEP55 in midbody/cytokinesis [46], [47] and HELLS in epigenetic DNA methylation and chromatin maintenance [48] further support the role of FOXM1B in genome maintenance [4]. We hypothesise that aberrant upregulation of FOXM1B may be inducing genomic instability through a program of malignant transformation involving deregulation of cytokinesis (CEP55) and epigenetic modifications (HELLS) whereby genomically unstable cells acquire oncogenic survival advantage (Fig. 8B).

In summary, this study provides, for the first time, several lines of evidence that FOXM1B is upregulated early during oral pre-malignancy and its expression persists in primary and metastatic HNSCC tissues. Cellular, molecular and genomic evidence have shown that nicotine, at concentrations relevant to tobacco chewers, significantly activated FOXM1 transcriptional activity in oral keratinocytes. The synergistic action of nicotine and FOXM1B overexpression led to anchorage-independent malignant transformation of a premalignant oral keratinocyte line which exhibited non-random genomic instability LOH/CNA profiles. Analysis of consensus LOH loci identified CEP55 and HELLS FOXM1B-target genes for malignant transformation. In conclusion, the potential of FOXM1B, CEP55 and HELLS as early oral cancer biomarkers deserves further investigation.

## Materials and Methods

### Samples

All clinical samples were collected according to local ethical committee-approved protocols and informed patient consent was obtained. Written informed consents and ethical approvals for all clinical samples used in this study were obtained from each institution where the samples were collected respectively. The present study involved 4 independent cohorts of patients' samples: 8 patients' fresh frozen oral tissues (Fig. 1A), 23 patients' primary keratinocyte cultures' microarray data [11] (Fig. 1B), 4 normal primary oral mucosa keratinocytes and 15 HNSCC derived primary cells were cultured as previously described [49] (Fig. 1C), and 25 patients' formalin-fixed paraffin embedded (FFPE) archival tissues (Fig. 1D–J).

### Cell culture

Primary normal human oral keratinocytes (NHOK1-5, 16, 355, 376, 881) were cultured in a modified version of Keratinocyte SFM (#17005-034 Gibco, Invitrogen) medium [50] containing 0.09 mM CaC1_2_. K-SFM was supplemented with 25 µg/ml bovine pituitary extract, 0.2 ng/ml EGF (#37000-015 Gibco, Invitrogen), and 1% penicillin/streptomycin. Oral premalignant cell lines: POE9n-hTERT/POE9n [50], DOK [51], D19, D20 and primary HNSCC derived cell lines: CA1, UK1, CaLH2, CaLH3, CaDec11, CaDec12, H357, 5PT, PE3/JA, VB6, and CaLH2-R [52] were kindly provided by Prof. Ian MacKenzie (ICMS, Centre of Cutaneous Research, Barts and The London School of Medicine & Dentistry). A non-transforming/premalignant Simian virus 40T (SV40T) antigen immortalised human buccal keratinocytes SVpgC2a [53] and a malignant human buccal squamous cell carcinoma line SqCC/Y1 [9] were both cultured in a low calcium (0.06 mM) EpiLife® keratinocyte growth medium (#M-EPI-500-CA; Cascade Biologics, TCS CellWorks Ltd., Buckinghamshire, UK.) with growth factor supplements (#S-001-5; Cascade Biologics). The malignant tongue-derived squamous cell carcinoma keratinocytes, SCC25 [12] were maintained in DMEM (Cambrex) supplemented with 10% FCS. Oral dysplasia and primary HNSCC derived cell lines were grown either in K-SFM, or RM^+^ medium consisting of DMEM supplemented with 25% Ham's F12 medium (Gibco, Invitrogen), 10% FCS, 1% penicillin/streptomycin (Invitrogen, Paisley, UK), and various mitogens (0.4 µg/ml hydrocortisone, 0.1 nM cholera toxin, 5 µg/ml transferring, 20 pM lyothyronine, 0.18 mM adenine, 5 µg/ml insulin and 10 ng/ml EGF). All cells were grown at 37°C in a humidified atmosphere of 10% (for DMEM and RM^+^) or 5% (for K-SFM and EpiLife) CO_2_/95% air.

### Bioinformatics analyses of microarray data

FOXM1 mRNA levels were analysed using published microarray data in various types of normal, premalignant and malignant human tissues. These microarray datasets were obtained from the Gene Expression Omnibus (GEO) database. Only studies that compared normal with premalignant and/or malignant tissues which used Affymetrix Gene Chip Human Genome U133A array (GEO platform GPL-96) were selected for analysis. The Affymetrix gene reference identification for FOXM1 was 202580_x_at. FOXM1 expression levels were statistically analysed using Student's t-test in Microsoft Excel software.

### Real-time absolute quantitative RT-PCR

Poly-A^+^ mRNA extraction, reverse transcription (RT) and real-time absolute quantitative RT-PCR (absolute copy number quantiation using standard curve for each gene) were performed using SYBR green I Master (hot-start Taq polymerase master mix) in the LightCycler 480 qPCR system (Roche Diagnostics Ltd, England, UK). FOXM1 isoform specific primers were designed to independently quantify each FOXM1 isoform. We have validated that these primer pairs are highly specific for each isoform without cross-amplification (see Supplemental Fig. S4). Common FOXM1 primers FOX-F 5′-ACTTTAAGCACATTGCCAAGC-3′ and FOX-R 5′-CGTGCAGGGAAAGGTTGT-3′ were used to detect all three FOXM1 isoforms. A panel of 8 human reference genes (HPRT1, UBC, GAPDH, RPLP0, ESD, 18SRNA, POLR2A and YAP1) [54], [55] were initially tested on a panel of human oral tissues used in this study (including normal gingival mucosa, normal buccal mucosa, normal tongue, dysplasias and HNSCC tissues), and keratinocyte cell types (including both primary and cell lines). Using the GeNorm algorithm method [55] and LightCycler 480 Relative Quantification Software (with built-in multiple reference genes normalisation algorithm), of the 8 reference genes, we identified the two most reliable and stable reference genes (POLR2A: POL-F, 5′-GCAAATTCACCAAGAGAGACG-3′; POL-R, 5′- CACGTCGACAGGAACATCAG-3′ and ESD: ESD-F, 5′-TCAGTCTGCTTCAGAACATGG-3′; ESD-R, 5′-CCTTTAATATTGCAGCCACGA-3′) across the aforementioned panel of human keratinocyte cells and tissues. POLR2A and ESD were used as reference genes for all subsequent qPCR experiments to calculate target gene expression levels.

### Plasmid construction

The retroviral vector pSIN-IP-GFP (Self-Inactivating-Internal Promoter-Green Fluorescence Protein [56] vector kindly provided by Dr. Paul Khavari (Stanford University School of Medicine, California, USA). The construction of pEGFP-FOXM1B, retroviral pSIN-EGFP-FOXM1B and the FOXM1-specific luciferase reporter pGL3-5BS was described previously [5]. We have previously validated that tagging of EGFP to the N-terminal of FOXM1B does not alter the functional activity of FOXM1B isoform [5]. Both EGFP and EGFP-FOXM1B transgenes are under the regulatory control of a CMV promoter. In some experiments, the pSIN-MCS (MCS) empty viral vector bearing no transgene was used as an additional control. In all experiments, transduction of MCS or EGFP did not produce any detectable biological artefacts. In this manuscript, the terms FOXM1B and EGFP-FOXM1B are synonymous and are used interchangeably to simplify descriptions.

### Retroviral transduction, transfection and FOXM1-specific transcriptional reporter assay

Retroviral supernatant was produced as previously described [5]. Briefly, cells to be retrovirally transduced were plated at 10∼40% confluence 24 hours prior to transduction. Cells were pre-incubated for 5 minutes in growth medium containing 5 µg/ml polybrene (hexadimethrine bromide; Sigma) before replacing with retroviral supernatant containing the same concentration of polybrene to facilitate infection. The cells in culture dishes with viral supernatant was then centrifuged (350×g) at 32°C for 1 hour before retroviral supernatant was replaced with normal growth medium and kept in normal culture condition. For transient transfection, cells were transfected using FuGENE 6 (Roche Biochemicals) and FOXM1 luciferase reporter assays were performed as described [5].

### RNA interference

We used an H1 promoter-driven hairpin siRNA vector (pSilencer 3.1-H1; Ambion Europe Ltd., Cambridgeshire, UK) to express shRNA against FOXM1 using a previously validated RNAi sequence [shFOX, CCTTTCCCTGCACGACATG [15]]. Keratinocytes were transfected with either shFOX or control shCtrl (containing a random sequence CATAGATAGGACATCAGCA which was validated to produce little off-target activity) using FuGENE 6 (Roche) according to manufacturer's protocol as described previously [57].

### Immunohistochemistry, fluorescence microscopy and digital pixel densitometry

FOXM1 was immunostained with a rabbit polyclonal antibody (K-19; Santa Cruz Biotechnology, California, USA) on paraffin sections as described [58]. The reaction product was visualised either with diaminobenzidine as a chromogenic substrate in FFPE tissue sections or with Alexa Fluor 488 goat anti-rabbit IgG secondary antibody (Molecular Probes, Invitrogen, Paisley, UK) for fluorescence microscopy imaging in cultured cells. As described previously [57], [59], digital pixel densitometry was performed using Adobe Photoshop CS3 (Adobe Systems Incorporated, USA) utilising the Colour Range tool for colour-based quantification or Lasso tool for manual selection of epithelial mucosa area and subsequent pixel area readout from the Histogram data chart. Whilst we have also used the ImageJ program for pixel densitometry and obtained similar results, Photoshop was the preferred choice for its accuracy, reproducibility and ease of use.

### Western blotting

Cells were lysed in 1× RIPA buffer [50 mM Tris 1 M, pH 7.3, 150 mM NaCl, 0.02% (w/v) SDS, 1% (v/v) Nonidet P-40, 100 mM sodium orthovanadate, 1× CM-EDTA complete mini EDTA-free cocktail tablets (Roche Diagnostic)]. Cell lysates were kept on ice for 20 min and centrifuged at 15000 rpm at 4°C for 20 min, and the supernatant was snap frozen in dry ice before being stored at −80°C. Protein concentration was measured using the Dc Protein Assay Kit (BioRad, Hertfordshire, UK). Proteins were extracted in 5× SDS sample buffer (1 M Tris-HCL, 2 M DTT, 1.5% Bromophenol Blue, 10% Glycerol, 20% SDS) and briefly denatured for 5 min at 100°C before loading on SDS-polyacrylamide gels. Proteins were then transferred to nitrocellulose membranes (Hybond C-extra; Amersham). Membranes were blocked in 5% non-fat dry milk in TBS (Tris buffered saline) containing 0.1% Tween-20 (Sigma) (TBST) for 30 min at room temperature. Next, membranes were probed with primary antibodies in either 3% TBST milk or 5% BSA (bovine serum albumin; Sigma) –TBST. Secondary antibodies were diluted in 3% TBST non fat dry milk. Antibodies used were rabbit polyclonal anti-FOXM1 (C-20, Santa Cruz Biotechnology), anti-β Tubulin (H235, Santa Cruz Biotechnology) and mouse monoclonal anti-β Catenin (Sigma). Secondary antibodies used were polyclonal rabbit anti-mouse immunoglobulin/HRP (DakoCytomation, Cambridgeshire, UK), polyclonal goat anti-rabbit immunoglobulin/HRP (DakoCytomation). Immunodetection was performed with enhanced chemiluminescence reagent ECL+ (Amersham Pharmacia). For protein dephosphorylation, cell lysates were incubated at 37°C for 1 hour in a mix containing 7.5 µg protein, 1× Protease Inhibitor Cocktail (Roche), 1× NEB buffer 3 and 1 unit/µg protein of calf-intestinal alkaline phosphatase (CIP; New England Biolabs). Sampels were then denatured for 5 minutes at 100°C and were run on 6% SDS PAGE and immunoblotted with the corresponding antibody.

### Cell viability and anchorage-independent cell transformation assay

CellTiter-Glo™ luminescent assay (Promega, Madison, WI, USA) was performed according to manufacturer's instruction to quantify ATP levels of metabolically active adherent cells. Nicotine/FOXM1B-transformed SVpgC2a cells (10^5^ cells) were plated in growth medium on top of a layer of solidified 1% agarose gel in tissue-culture plates. Culture medium was changed twice weekly. During medium change, floating cells were collected by centrifugation and re-introduced into the agarose dishes containing fresh medium. After 3 weeks, surviving colonies (only FOXM1B-expressing cells survived the colony formation assay; Fig. 4H) were individually aspirated using 200 µl pipette tips from the agar dishes and subsequently propagated into individual transformed clonal lines (SVFN1-8).

### SNP microarray mapping assay

Genomic DNA (gDNA) samples were purified and processed as described previously [60]–[62] with slight modification to the standard GeneChip Mapping 10 K (V2.0) Xba Assay protocol (Affymetrix Inc., Santa Clara, CA). Briefly, 350–500 ng of DNA was digested with XbaI and ligated to the XbaI adaptor prior to PCR amplification using AmpliTaq Gold with Buffer II (Applied Biosystems, Foster City, CA). The PCR amplication profile on an MJ DNA Engine thermal cycler was: 95/180 (hot start) and 95/20, 59/15, 72/60 (°C/sec) for 40 cycles followed by a termination step at 72°C for 7 mins. PCR products were purified using Ultrafree-MC 30,000 NMWL filter columns (Cat#UFC-3LTK00; Amicon) and DNA concentrations determined using NanoDrop spectrophotometer. Hybridisation and scanning of the arrays were processed as described previously [60]–[62]. LOH and LOH likelihood were analyzed using Affymetrix Copy Number Analysis Tool software (CNAT, version 4) where the threshold for statistical significance was P≤10-6 as recommended by Affymetrix [63], [64]. The data was cross verified and genome ploidy ±standard deviation for Log 2 ratio values were obtained using an independent algorithm, Copy Number Analyzer for GeneChip (CNAG, version 2), which has been shown to improve signal-to-noise ratios in heterogeneous genomes [65]. Average SNP call rates for all chips were 95–99%. Putative genes within or adjacent to the LOH SNPs were identified using IdeogramBrowser (version 0.20.0) [66] based on NCBI Human Genome Assembly (Built 36.2 database). Raw SNP data can be accessed from the NCBI's GEO database (GEO accession: GSE11782).

### Chromatin immunoprecipitation qPCR (ChIP-qPCR)

ChIP was performed using the ChampionChIP™ One-Day Kit for qPCR (#1035A; SABiosciences Corporation, Frederick, MD, USA) according to manufacturer's protocol. To ensure that the assay is properly controlled and specific, ChIP was performed using anti-GAPDH, anti-cMYC or anti-FOXM1 antibodies each on either mock, EGFP or FOXM1B-transduced keratinocytes. Putative FOXM1 DNA binding sites [67] within promoter regions (within 1 Kb upstream of gene) of CEP55 and HELLS were identified using ClustalW2 sequence alignment tool [68]) and qPCR primers flanking the putative binding site were designed (Supplemental Fig. S9). Absolute qPCR was performed using the LightCycler 480 qPCR system as described above.

## Supporting Information

Figure S1 Upregulation of FOXM1 protein in oral premalignant and HNSCC cell lines. Immunofluorescence imaging of FOXM1 (FITC, green) and DNA (DAPI, blue) in normal (NHOK1, HOK365), dysplasia (D20, POE9n) and HNSCC lines (5PT, UK1).
(3.37 MB TIF)

Figure S2 Upregulation of FOXM1B protected SVpgC2a cells from nicotine-induced cell death. (A) Fluorescence microscopy showing green fluorescence expression of EGPF or EGFP-FOXM1B expressing SVpgC2a, SqCC/Y1 and SCC25 cells, either treated with vehicle control (H20) or nicotine (10 mM) for 24 hours prior to experiment. Corresponding brightfield images show respective cell density. (B) Digital densitometry quantification of images in A showed that nicotine induced significant cell death in EGFP-expressing SVpgC2a cells but not in FOXM1B-expressing cells. Nicotine treatment did not show toxic effect on SqCC/Y1 or SCC25 cells.
(1.52 MB TIF)

Figure S3 FOXM1B overexpression alone in the premalignant oral keratinocyte SVpgC2a cells induces LOH in chromosome 13. Magnified view of LOH profiles in chromosome 13 across the 8 SVFN transformed clones with a list of randomly selected genes found within the consensus LOH loci in this chromosome.
(8.00 MB TIF)

Figure S4 FOXM1-isoform specific real-time quantitative RT-PCR (qPCR). (A) A schematic diagram showing the relative location of primers specific to each FOXM1 isoform. (B) Primer sequences. (C) qPCR amplification curves demonstrating the specificity of each isoform-specific primer pairs in the presence of different FOXM1 isoform DNA templates (106 copies were added in each qPCR reaction). These primer pairs only amplified specific FOXM1 isoform and showed no cross-amplification in the presence of inappropriate FOXM1 isoform templates. (D) Melting analysis showed a single PCR product amplified by each pair of primers respectively. (E) Agarose-gel electrophoresis confirmed the correct PCR product size respective to each isoform. These PCR products were subsequently validated by nucleotide sequencing analysis (data not shown).
(0.89 MB TIF)

Figure S5 Bioinformatics analysis of CEP55 gene expression level from a published microarray dataset (GEO accession: GDS1477) comparing BT-20 breast cancer cells expressing either mock, siGFP or siFOX at 48 h post-transfection (Wonsey and Follettie, 2005).
(0.61 MB TIF)

Figure S6 Bioinformatics analysis of FOXM1, CEP55 and HELLS gene expression level from a published microarray dataset (GEO accession: GDS1548). Analysis of oral squamous cell carcinoma (OSCC) cells from 16 patients and 4 healthy normal oral tissue samples. OSCC cells were isolated from tumours by laser capture microdissection. Results identify a strong correlation between FOXM1 (A), CEP55 (B) and HELLS (C) gene expression profiles and tumor invasiveness in OSCC. (D) Linear regression analysis showed that CEP55 expression is highly correlated (R2 = 0.8037) with FOXM1. (E) HELLS expression correlated (R2 = 0.6132) less significantly with FOXM1. (F) CEP55 and HELLS expressions showed poor correlation (R2 = 0.5289) with each other.
(1.02 MB TIF)

Figure S7 ChIP-qPCR for promoters of CEP55 and HELLS. Representative qPCR fluorescence curves and melting analysis of the promoters of CEP55 (A) and HELLS (B) on ChIP (immunoprecipitate/IP; with either anti-GAPDH, anti-cMYC or anti-FOXM1) and input (non-IP) fractions on mock, EGFP or FOXM1B-transduced oral keratinocytes.
(0.54 MB TIF)

Figure S8 ChIP-qPCR data for promoters of cyclinB1/CCNB1, VEGF and p16/CDKN2A. Quantitative PCR data showing the relative copy number of promoters of CCNB1 (A), VEGF (B–C, promoter region 1 and 2) and CDKN2A (D) in the three ChIP fractions (anti-GAPDH, anti-cMYC or anti-FOXM1) on mock, EGFP or FOXM1B-transduced oral keratinocytes. *P<0.05, **P<0.01 and ***P<0.001 indicate t-test significant levels.
(0.24 MB TIF)

Figure S9 Primer sequences for ChIP-qPCR. qPCR primers were designed within 1 kilobase upstream from +1 of each gene. For CEP55 and HELLS promoters, primers were designed to encompass putative FOXM1 binding site located using ClustalW2 sequence alignment tool (see Methods).
(0.25 MB TIF)
