# Supplementary figures and images for: FOXM1 Upregulation Is an Early Event in Human Squamous Cell Carcinoma and it Is Enhanced by Nicotine during Malignant Transformation
Source: PLoS One. 2009 Mar 16;4(3):e4849. doi: 10.1371/journal.pone.0004849 (PMC2654098; doi:10.1371/journal.pone.0004849)

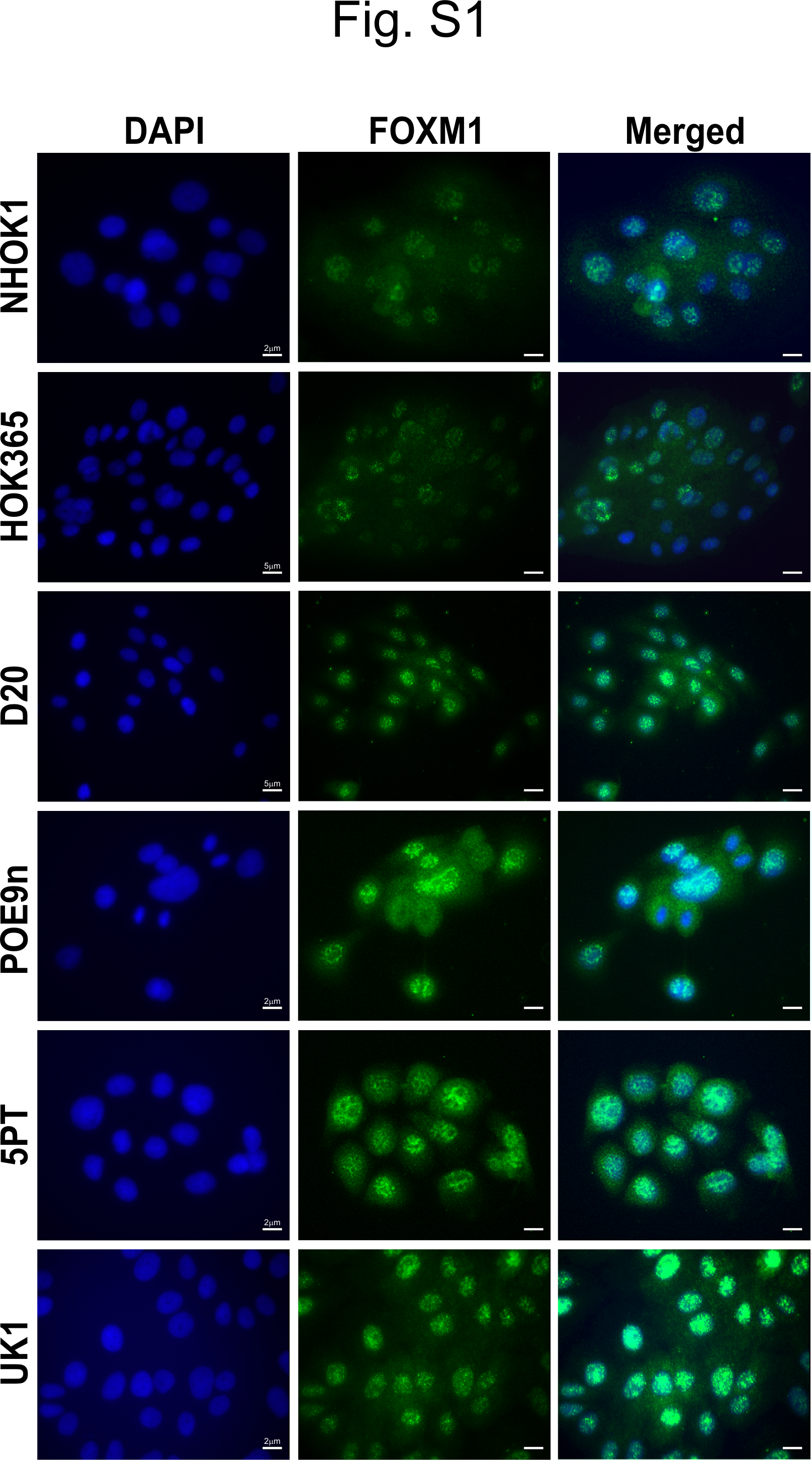

Supplement: Figure S1 — Upregulation of FOXM1 protein in oral premalignant and HNSCC cell lines. Immunofluorescence imaging of FOXM1 (FITC, green) and DNA (DAPI, blue) in normal (NHOK1, HOK365), dysplasia (D20, POE9n) and HNSCC lines (5PT, UK1). (3.37 MB TIF) [file pone.0004849.s001.tif]

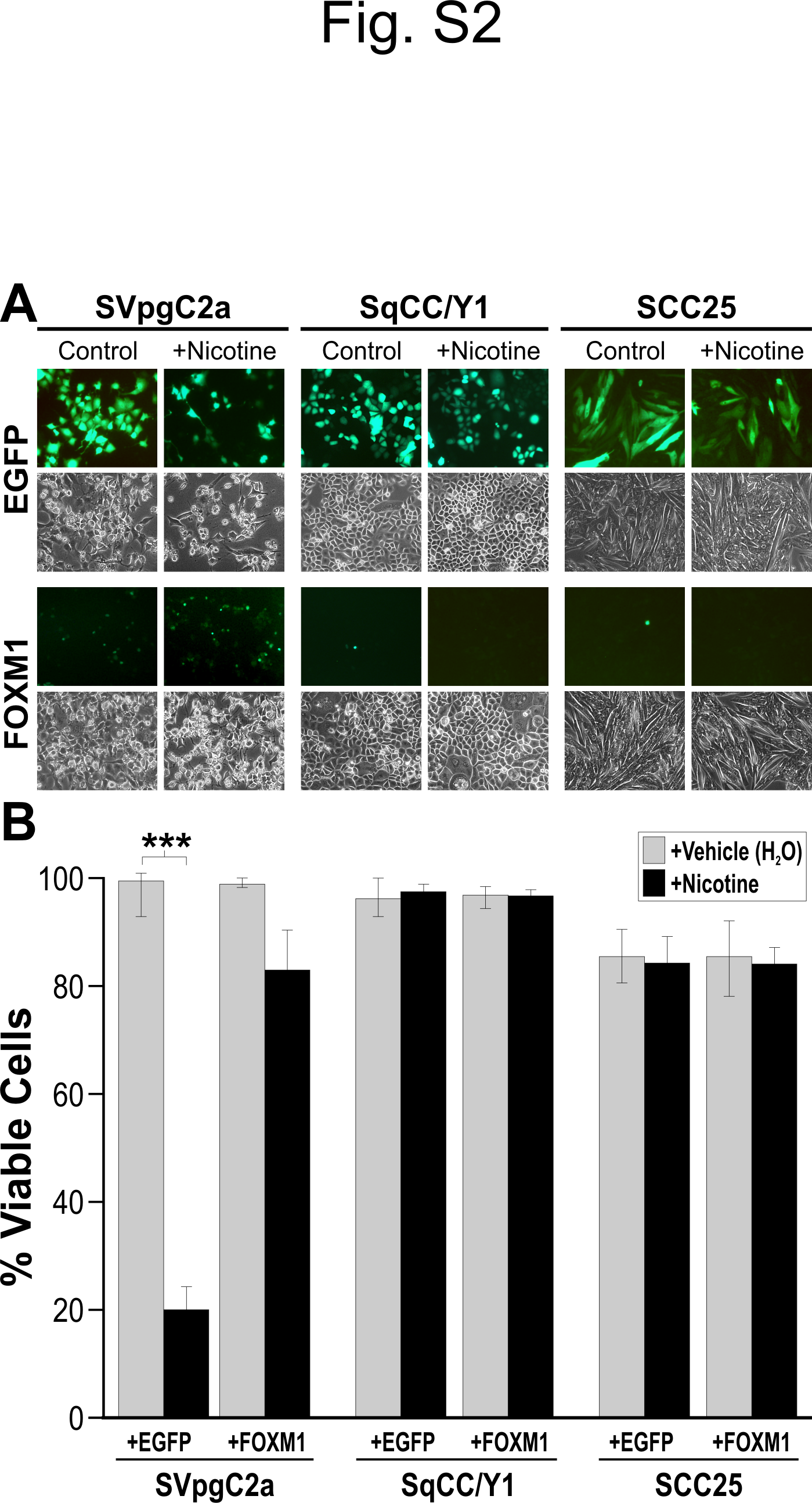

Supplement: Figure S2 — Upregulation of FOXM1B protected SVpgC2a cells from nicotine-induced cell death. (A) Fluorescence microscopy showing green fluorescence expression of EGPF or EGFP-FOXM1B expressing SVpgC2a, SqCC/Y1 and SCC25 cells, either treated with vehicle control (H20) or nicotine (10 mM) for 24 hours prior to experiment. Corresponding brightfield images show respective cell density. (B) Digital densitometry quantification of images in A showed that nicotine induced significant cell death in EGFP-expressing SVpgC2a cells but not in FOXM1B-expressing cells. Nicotine treatment did not show toxic effect on SqCC/Y1 or SCC25 cells. (1.52 MB TIF) [file pone.0004849.s002.tif]

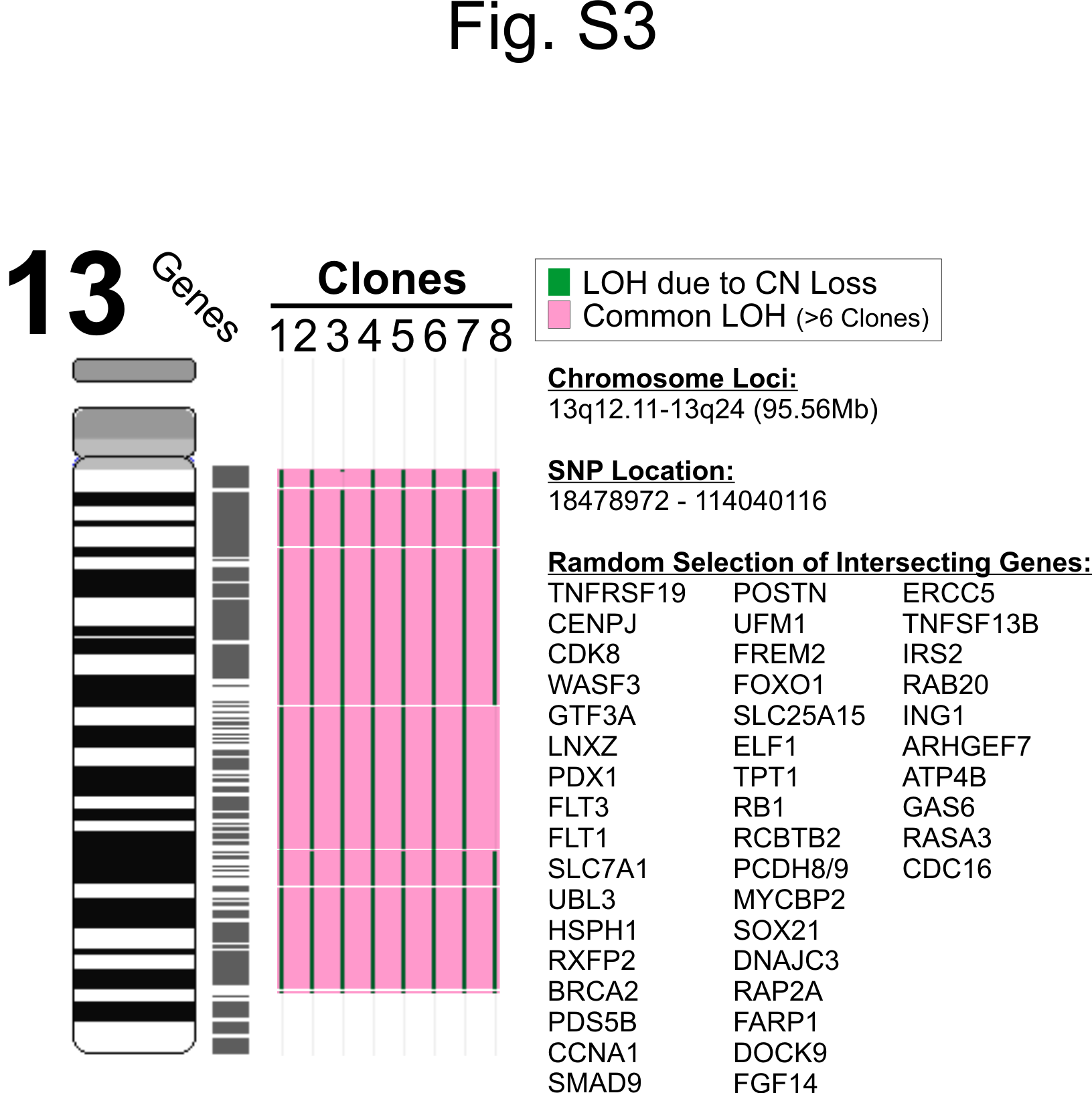

Supplement: Figure S3 — FOXM1B overexpression alone in the premalignant oral keratinocyte SVpgC2a cells induces LOH in chromosome 13. Magnified view of LOH profiles in chromosome 13 across the 8 SVFN transformed clones with a list of randomly selected genes found within the consensus LOH loci in this chromosome. (8.00 MB TIF) [file pone.0004849.s003.tif]

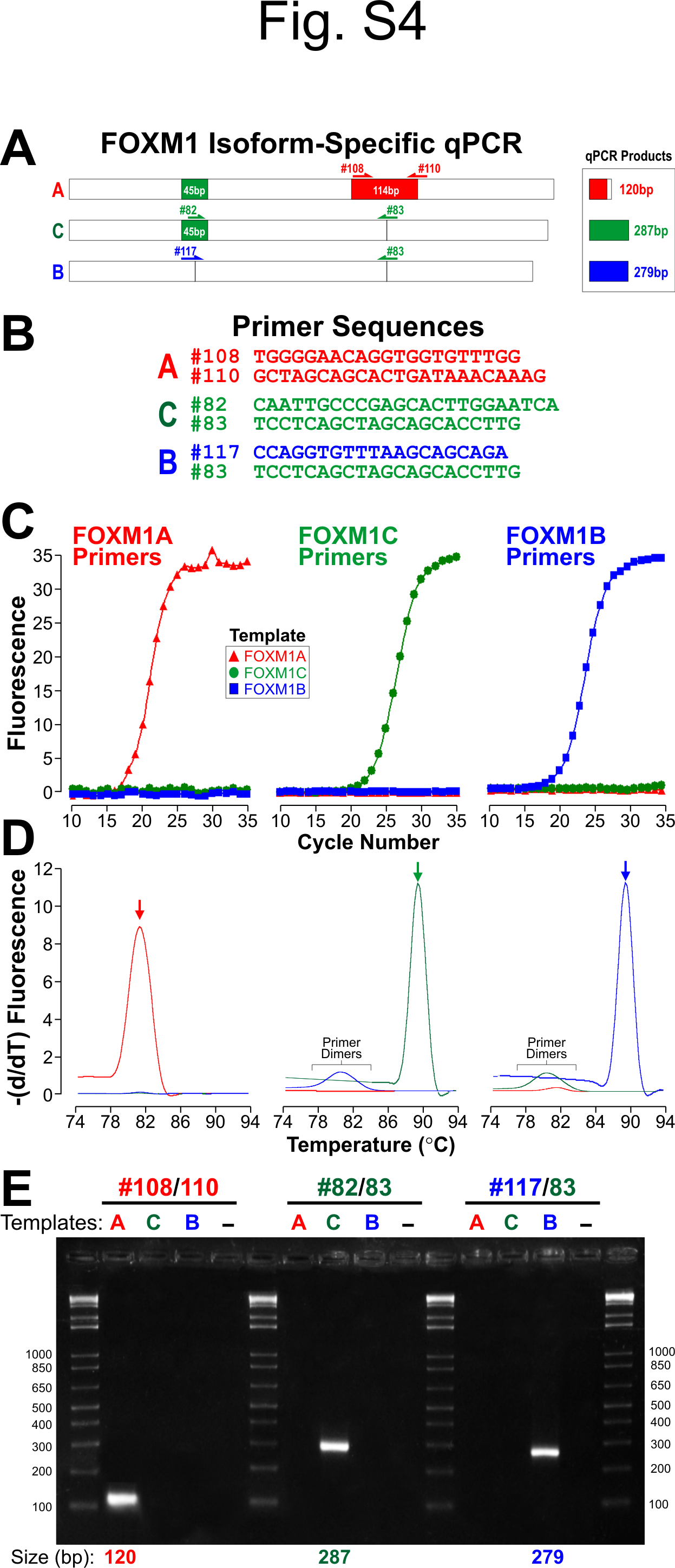

Supplement: Figure S4 — FOXM1-isoform specific real-time quantitative RT-PCR (qPCR). (A) A schematic diagram showing the relative location of primers specific to each FOXM1 isoform. (B) Primer sequences. (C) qPCR amplification curves demonstrating the specificity of each isoform-specific primer pairs in the presence of different FOXM1 isoform DNA templates (106 copies were added in each qPCR reaction). These primer pairs only amplified specific FOXM1 isoform and showed no cross-amplification in the presence of inappropriate FOXM1 isoform templates. (D) Melting analysis showed a single PCR product amplified by each pair of primers respectively. (E) Agarose-gel electrophoresis confirmed the correct PCR product size respective to each isoform. These PCR products were subsequently validated by nucleotide sequencing analysis (data not shown). (0.89 MB TIF) [file pone.0004849.s004.tif]

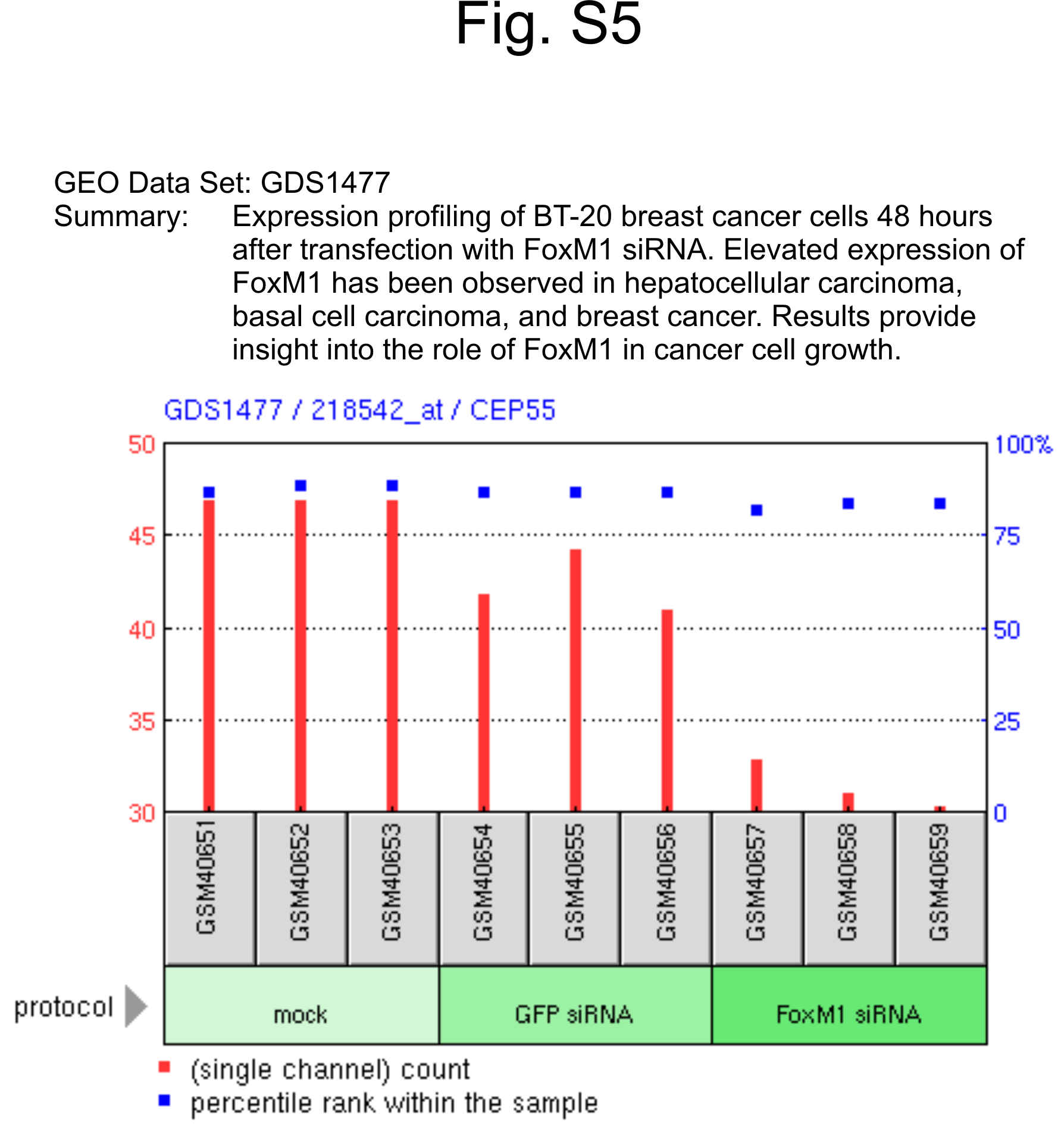

Supplement: Figure S5 — Bioinformatics analysis of CEP55 gene expression level from a published microarray dataset (GEO accession: GDS1477) comparing BT-20 breast cancer cells expressing either mock, siGFP or siFOX at 48 h post-transfection (Wonsey and Follettie, 2005). (0.61 MB TIF) [file pone.0004849.s005.tif]

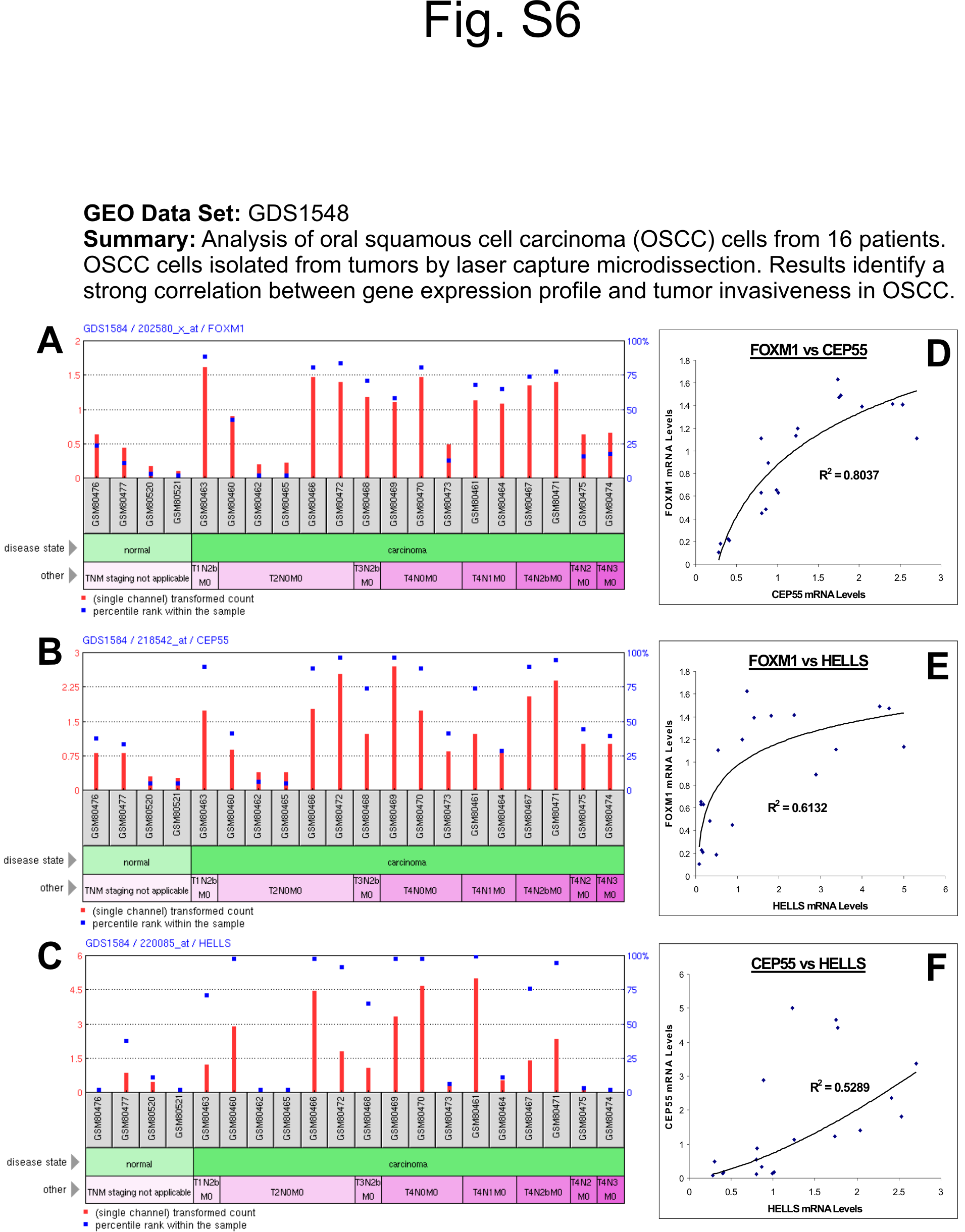

Supplement: Figure S6 — Bioinformatics analysis of FOXM1, CEP55 and HELLS gene expression level from a published microarray dataset (GEO accession: GDS1548). Analysis of oral squamous cell carcinoma (OSCC) cells from 16 patients and 4 healthy normal oral tissue samples. OSCC cells were isolated from tumours by laser capture microdissection. Results identify a strong correlation between FOXM1 (A), CEP55 (B) and HELLS (C) gene expression profiles and tumor invasiveness in OSCC. (D) Linear regression analysis showed that CEP55 expression is highly correlated (R2 = 0.8037) with FOXM1. (E) HELLS expression correlated (R2 = 0.6132) less significantly with FOXM1. (F) CEP55 and HELLS expressions showed poor correlation (R2 = 0.5289) with each other. (1.02 MB TIF) [file pone.0004849.s006.tif]

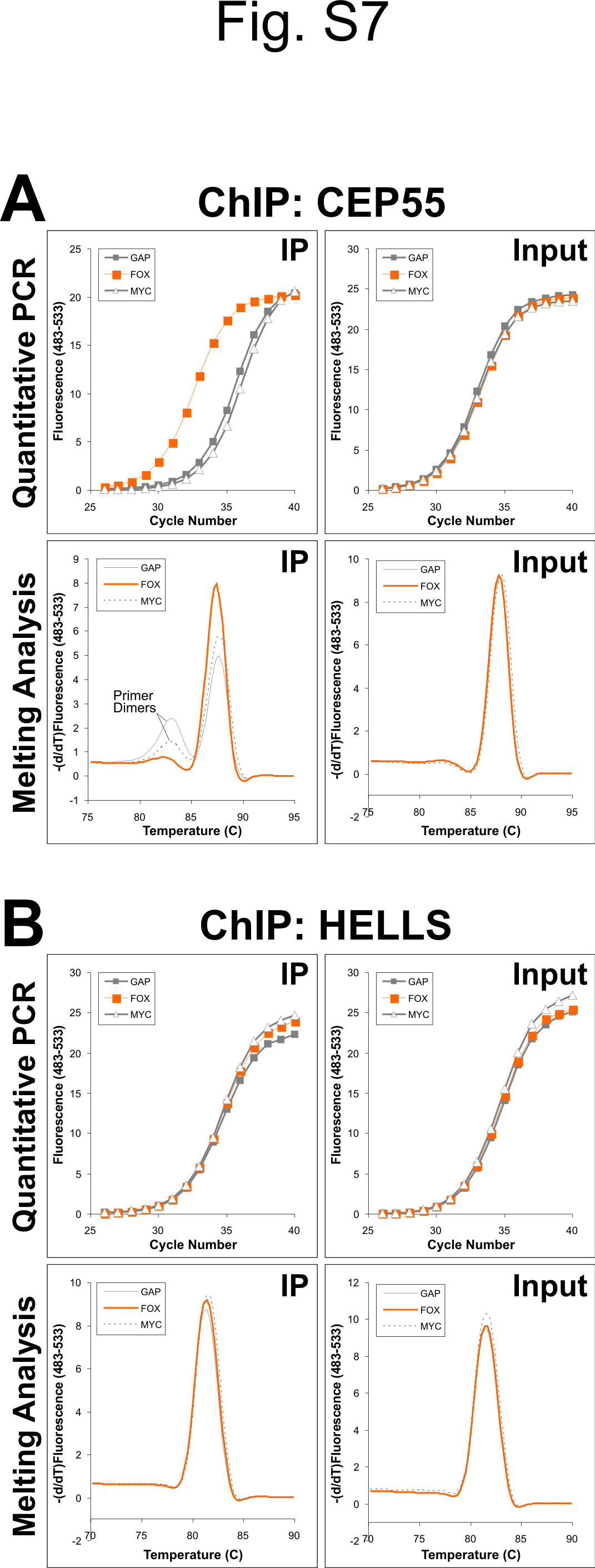

Supplement: Figure S7 — ChIP-qPCR for promoters of CEP55 and HELLS. Representative qPCR fluorescence curves and melting analysis of the promoters of CEP55 (A) and HELLS (B) on ChIP (immunoprecipitate/IP; with either anti-GAPDH, anti-cMYC or anti-FOXM1) and input (non-IP) fractions on mock, EGFP or FOXM1B-transduced oral keratinocytes. (0.54 MB TIF) [file pone.0004849.s007.tif]

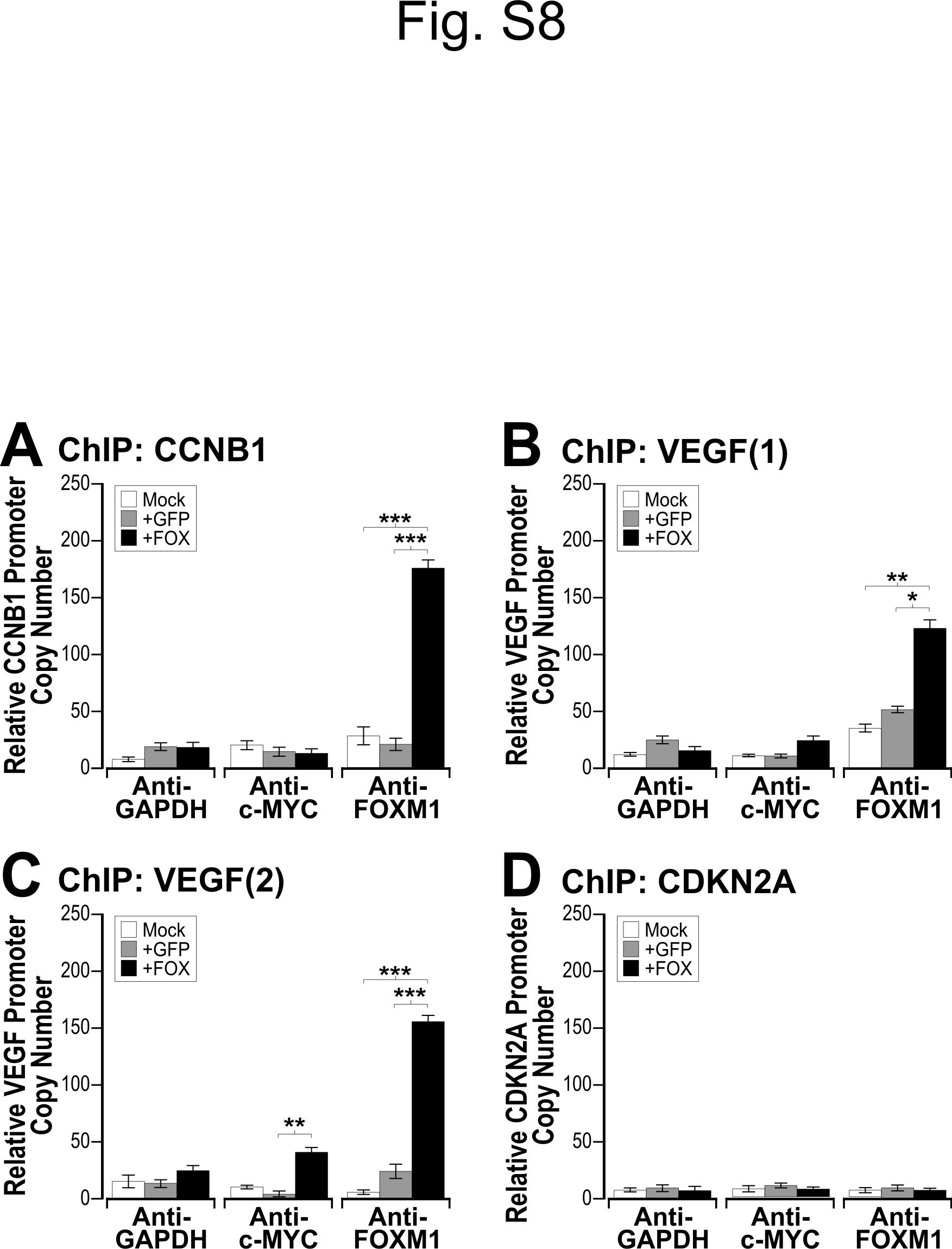

Supplement: Figure S8 — ChIP-qPCR data for promoters of cyclinB1/CCNB1, VEGF and p16/CDKN2A. Quantitative PCR data showing the relative copy number of promoters of CCNB1 (A), VEGF (B–C, promoter region 1 and 2) and CDKN2A (D) in the three ChIP fractions (anti-GAPDH, anti-cMYC or anti-FOXM1) on mock, EGFP or FOXM1B-transduced oral keratinocytes. *P<0.05, **P<0.01 and ***P<0.001 indicate t-test significant levels. (0.24 MB TIF) [file pone.0004849.s008.tif]

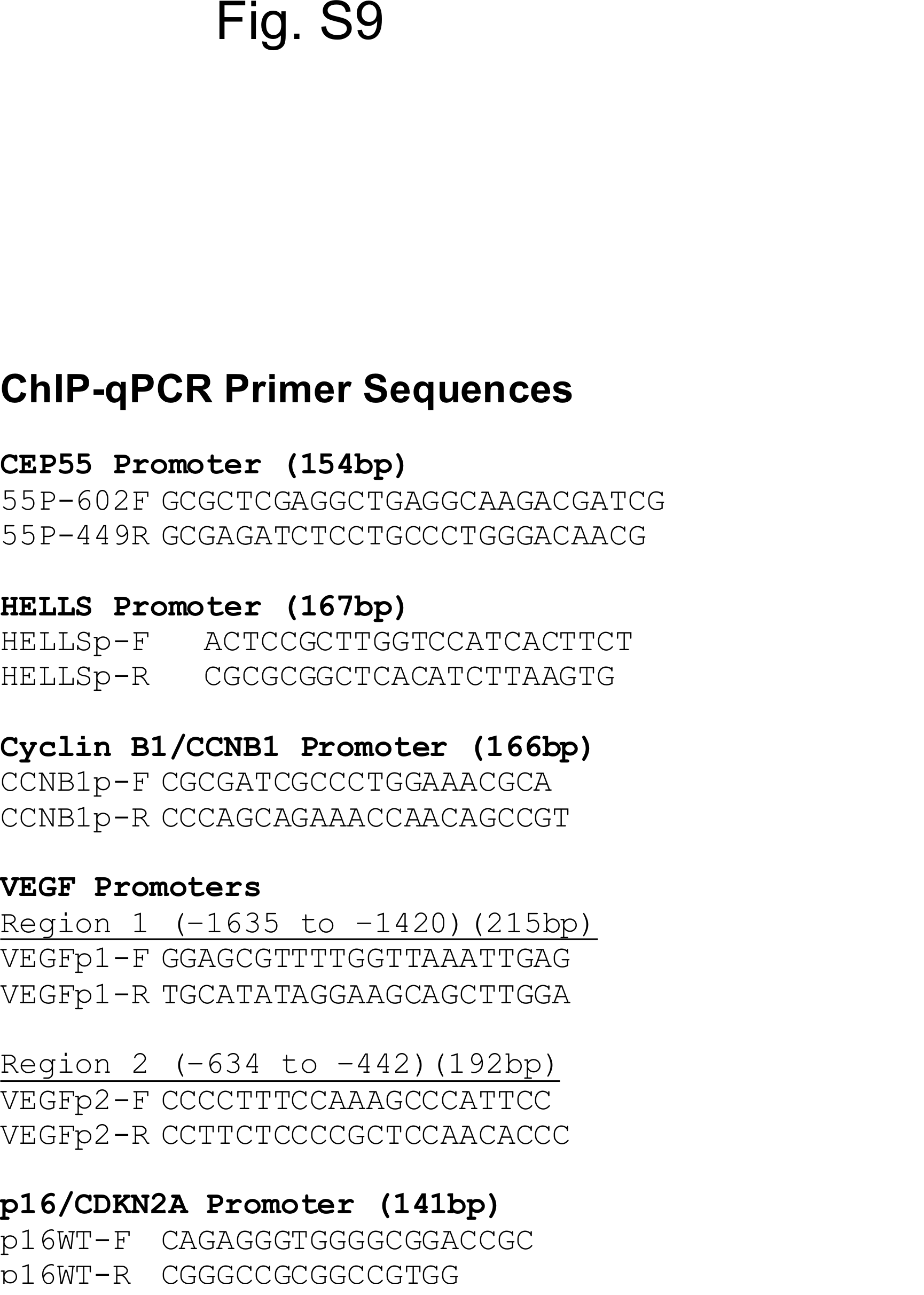

Supplement: Figure S9 — Primer sequences for ChIP-qPCR. qPCR primers were designed within 1 kilobase upstream from +1 of each gene. For CEP55 and HELLS promoters, primers were designed to encompass putative FOXM1 binding site located using ClustalW2 sequence alignment tool (see Methods). (0.25 MB TIF) [file pone.0004849.s009.tif]
